# Unsupervised characterization of 100,272 EHR patients identifies high-risk groups and comorbidities linked to premature aging

**DOI:** 10.1038/s41746-026-02913-x

**Published:** 2026-06-23

**Authors:** Su Xian, Jordan W. Smoller, Yuan Luo, Theresa L. Walunas, Cong Liu, Atlas Khan, Chunhua Weng, Iftikhar J. Kullo, Wei-Qi Wei, Gail P. Jarvik, David R. Crosslin

**Affiliations:** 1 Department of Biomedical Informatics and Medical Education, University of Washington, Seattle, WA USA; 2 Psychiatric and Neurodevelopmental Genetics Unit, Center for Genomic Medicine, Massachusetts General Hospital, Boston, MA USA; 3 Department of Psychiatry, Center for Precision Psychiatry, Massachusetts General Hospital, Boston, MA USA; 4 Department of Preventive Medicine, Northwestern University Feinberg School of Medicine, Chicago, IL USA; 5 Department of Medicine, Northwestern University Feinberg School of Medicine, Chicago, IL USA; 6 Department of Pediatrics, Division of Genetics and Genomics, Boston Children’s Hospital, Boston, MA USA; 7 Department of Medicine, Columbia University Irving Medical Center, Columbia University, New York, NY USA; 8 Department of Biomedical Informatics, Columbia University, New York, NY USA; 9 Department of Cardiovascular Medicine and the Gonda Vascular Center, Mayo Clinic Rochester Minnesota, Rochester, MN USA; 10 Department of Biomedical Informatics, Vanderbilt University Medical Center, Nashville, TN USA; 11 Department of Medicine, Division of Medical Genetics, University of Washington, Seattle, WA USA; 12 Department of Genome Sciences, University of Washington, Seattle, WA USA; 13 Department of Medicine, Division of Biomedical Informatics and Genomics, Tulane University, New Orleans, LA USA

**Keywords:** Biomarkers, Computational biology and bioinformatics, Diseases, Genetics, Medical research, Risk factors

## Abstract

Electronic health records (EHRs) contain extensive multidimensional patient data, presenting challenges for the discovery of novel and meaningful clinical patterns. Unsupervised clustering of high-dimensional clinical data holds great potential for identifying novel clinical patterns. Here, we performed unsupervised clustering and characterized 100,272 patients in the Electronic Medical Records and GEnomics (eMERGE) Network. We identified 70 clusters defined by distinct comorbidity patterns. Meanwhile, age and sex are also strongly associated with patient stratification, influencing phenotype prevalence and onset time. Notably, phenotype onset time accurately predicted chronological age and was significantly associated with overall mortality risk. Besides age and sex, we assessed the contribution of genetic variation to phenotype development and observed evidence of cross-phenotype associations influencing cluster membership and comorbidity patterns. However, the role of genetics recedes during aging. We also identified several high-risk clusters with elevated Charlson Comorbidity Index (CCI) scores and validated these findings in an independent cohort. Further analysis of these clusters revealed phenotypes linked to premature aging and highlighted a survival selection among older participants in observational studies. Overall, this study enables phenome-wide unsupervised patient stratification for multimorbidity discovery in largely unannotated clinical data, offering valuable insights into patient stratification, comorbidity analysis, aging, and health outcomes.

## Introduction

Electronic health records (EHRs) have great potential for revolutionizing healthcare delivery and research by providing comprehensive data on patient demographics, clinical diagnoses, laboratory results, medications, and procedures^1–3^. This rich source of real-world data offers unprecedented opportunities for advancing medical research and improving patient care. Researchers can leverage EHRs to explore health trends, phenotype patients, study disease mechanisms, and develop evidence-based interventions^2,4–6^. In the current era, novel machine learning algorithms are actively involved in the study of phenotypes, clinical presentations, and response patterns^2,7^. For example, machine learning techniques are increasingly employed to extract meaningful phenotypic traits, enabling the identification of novel disease subtypes^8–10^. However, fully harnessing the potential of EHR data to characterize disease patterns and uncover trends among patient populations remains a significant challenge. The transformation of vast and complex EHR datasets into actionable insights is essential for advancing disease monitoring and predictive clinical tasks. Unsupervised learning methods are particularly well-suited to this objective, as they enable the meaningful grouping of similar patients and reveal previously unexplored patterns underlying diverse disease subtypes.

Data representation methods, a type of unsupervised learning approach, including matrix decomposition, Latent Dirichlet Allocation, and modern neural network approaches such as autoencoders, have been increasingly used to uncover complex relationships and latent structures between features in EHR data^11–13^. These approaches offer not only dimension reduction by casting data to the latent space, but also enable the discovery of latent data structures that do not rely on prior domain knowledge^14–17^. After dimension reduction and data representation, there is a need to summarize and characterize patterns from the latent space. Topic modeling, originally developed in the field of natural language processing, is a statistical technique that identifies latent themes in large collections of text. Topic modeling classifies documents by the mixtures of topics, where a distribution over words represents each topic. Through topic modeling, one can obtain a distributional summary of documents from their topic words and further categorize the documents. Researchers have demonstrated the potential of topic modeling in a wide range of applications and data analysis^18,19^. Thus, topic modeling can serve as an appropriate unsupervised data summary approach.

Recent advances in human biology and medicine have enabled the discovery and characterization of numerous health-related phenotypes^20–22^. Especially in the era of big data, there lies great potential to identify unknown clinical patterns and uncover novel clusters of comorbidities within EHR data using unsupervised analysis to jointly study patient phenotypes^8,9,23,24^. Here, we conducted a large-scale analysis of EHR data to explore the interplay among phenotypes, genetics, and demographic factors. We started with an unsupervised clustering approach to group patients into clusters and then utilized topic analysis to characterize phenotypic clusters. We found that each patient cluster is composed of a unique combination of phenotypes and shows strong patterns related to age and sex. Among these clusters, age and sex, along with genetics, are the primary factors influencing patient stratification and phenotype development. Yet the contribution of genetics fades out when age increases. Importantly, patient age is a crucial determinant of the phenotypes they harbor; the phenotypes, in turn, accurately predict patient age. At the same time, the onset time and prevalence of phenotypes are drastically different between females and males. When examining health outcomes, we identified specific clusters with elevated mortality risk and characterized their comorbidity patterns. Notably, we discovered that the onset age of 82 phenotypes was significantly associated with mortality in two independent cohorts, with essential hypertension showing the strongest effect, suggesting a link to premature aging. Building on this, we uncovered evidence of survival selection and further characterized individuals who had aged successfully. Together, this work demonstrates the potential of EHR-based unsupervised learning for revealing hidden clinical patterns, advancing patient stratification, and deepening our understanding of aging and disease progression.

## Results

### Unsupervised clustering of 100,272 patients in the eMERGE cohort

**Fig. 1:**
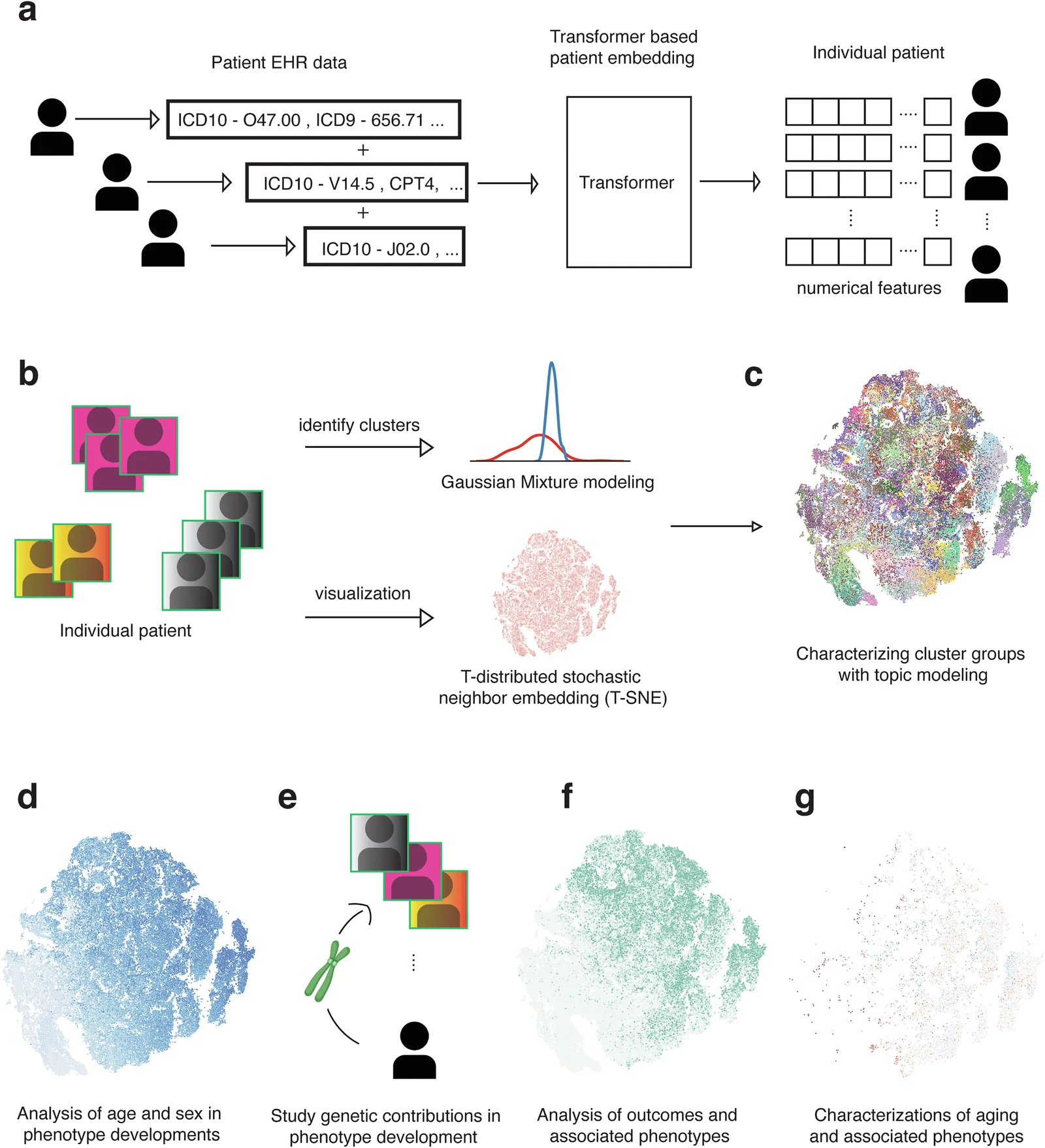
Illustration of the study framework using 100,272 eMERGE participants EHR data. **a** Illustration of extracting numerical embeddings from a pre-trained patient embedding model as a representation of digital phenotypes. **b** Illustration of patient clustering using a Gaussian Mixture model yielding 70 clusters of patients and visualized using T-SNE in two dimensional. **c** Topic analysis to characterize patient clusters with distinct comorbidity patterns. **d** Analyzing clusters and phenotype associations with age and sex. **e** Analyzing cross-phenotype association in cluster groups. **f** Investigating mortality rate differences in cluster groups and identifying phenotypes associated with premature aging. **g** Investigation of survival selection and characterizing aging using longitudinal data.

We applied an unsupervised clustering approach to jointly analyze EHR data from 100,272 participants in the eMERGE network, using transformer-based patient embeddings (Fig. 1a, b) to uncover novel clinical patterns and emerging health trends^25^. In brief, we utilized a transformer-based embedding model to represent patients’ diagnosis and procedure patterns as numerical vectors, referred to hereafter as patient embeddings^10^. The model is trained to predict diagnoses and procedures occurring in the following year based on those observed in the current year, thereby learning patterns of disease co-occurrence and transition at the event level. Importantly, the model does not incorporate demographic or patient-level information, such as age, sex, or patient identity, during training. Detailed descriptions of the model architecture, hyperparameters, and training procedure are provided in our previous work^10^. We applied the Gaussian Mixture Model (GMM) to cluster the numerically embedded patients (Fig. 1b) and visualized each patient as a data point in two dimensions (Fig. 1b) using t-distributed stochastic neighbor embedding (TSNE). The optimal number of clusters and clustering methods are selected based on evaluation of adjusted mutual information (AMI), Bayesian Information Criteria (BIC), clinical interpretability, and cluster distinctiveness (“Methods”). While 15 or 30 clusters would have been statistically favored under BIC and AMI, this would collapse clinically distinct subgroups, and 70 clusters were selected to preserve interpretability at a minor stability cost (AMI 0.74 to 0.68). This study follows a multi-step analytical framework. We first employed a topic modeling strategy to characterize the comorbidity pattern of individual clusters (Fig. 1c). We then studied the impact of sex and age on cluster membership, phenotype prevalence, and onset timing (Fig. 1d). Next, we investigated genetic contributions to phenotype developments and discovered cross-phenotype associations within individual clusters (Fig. 1e). Importantly, we then identified clusters associated with elevated rates of mortality and characterized premature aging associated phenotypes (Fig. 1f). Lastly, we uncovered evidence of survival selection in observational studies and characterized successful aging populations, highlighting their association with the delayed onset of aging-related phenotypes (Fig. 1g).

### Topic analysis stratifies patients with unique phenotype patterns

**Fig. 2:**
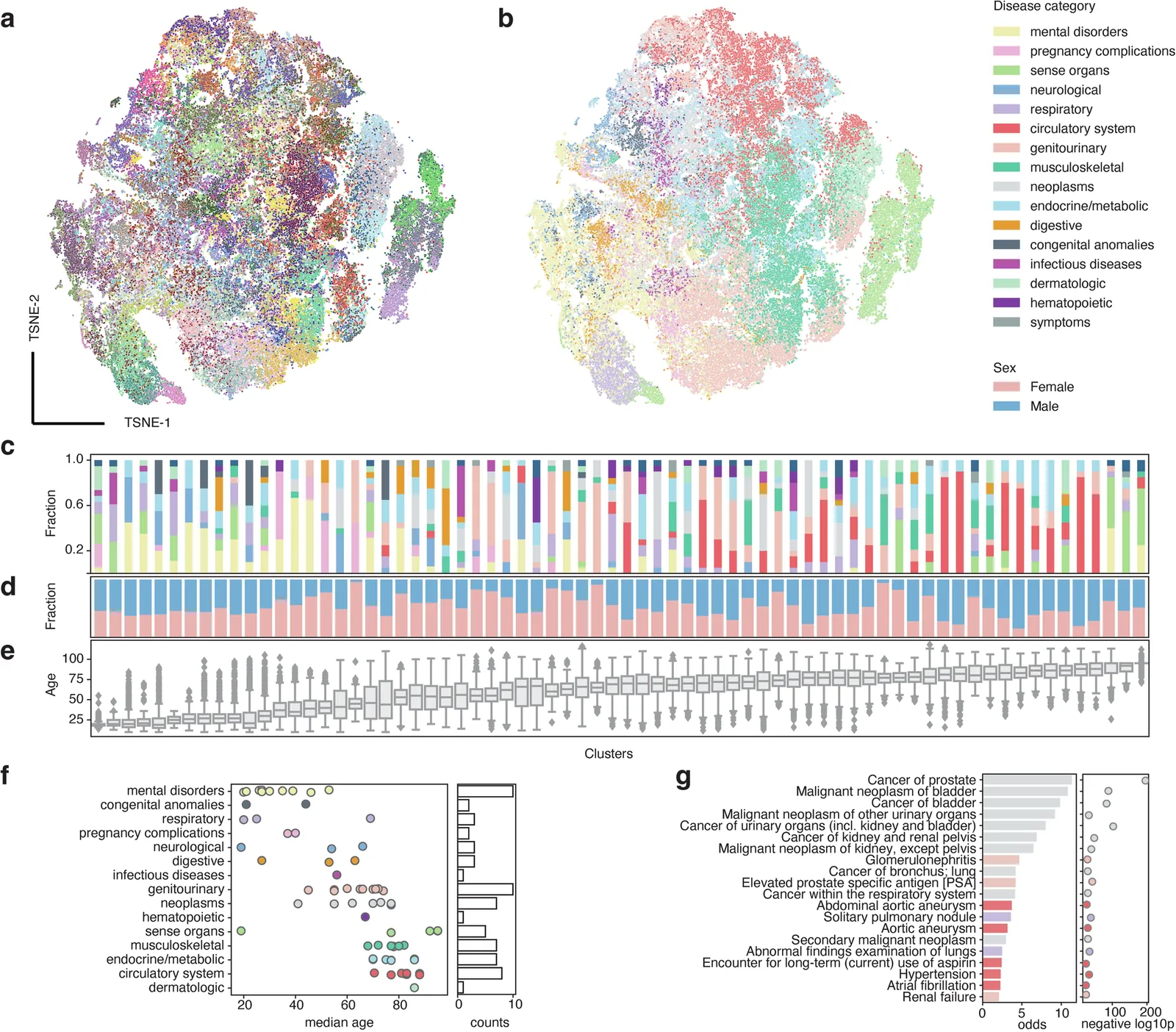
Characterizing topics for each patient cluster. **a** Scatterplot showing T-SNE visualizations of 100,272 patients from the eMERGE cohort, colored by 70 unique cluster groups, with each dot representing a patient. **b** Scatterplot of the same T-SNE visualizations from **a** but colored by their topics defined by phecode category. **c** Barplot showing the fractions of the top five phenotype categories in each cluster group. **d** Barplot showing Fraction of female and male ratio in each cluster group. **e** Boxplot showing the distribution of age in each cluster group. **f** Stripplot showing the median age onset of the corresponding topic (left) and the number of times the specific topic occurred in the eMERGE cohort (right). **g** Barplot represents an example of the top 20 enriched phenotypes in cluster 47 (left) and their negative log10 *p*-values (right), showing in a stripplot.

The GMM grouped patients into 70 unique clusters represented by a complex visual structure (Fig. 2a), with each cluster group comprising hundreds to thousands of patients. To deliver a meaningful summary for each cluster, we examined the top 20 enriched phenotypes (defined by phecodes) in each cluster, quantified the most frequent phenotype category as the main topic for each cluster (“Methods”). As shown in Fig. 2, we observed a clear and meaningful clustering of phenotype categories (Fig. 2a, b). Each cluster harbored a uniquely enriched combination of phenotypes, though all were associated with age and sex (Fig. 2c–e). For example, mental disorders (light yellow) are common in clusters enriched with younger participants (Fig. 2c). As participant age increased, circulatory system diseases (dark purple) became more prominent. This age related effect is more evident in the graph of median onset time of each cluster topic phenotype, shown in Fig. 2f, where mental disorders are prevalent topics among youth and middle age; pregnancy complications appear in two clusters with a median age of around 40; genitourinary and neoplasms are more common in clusters of age from 40 to 80; while musculoskeletal, endocrine/metabolic, circulatory system were predominant in clusters with older patients (median age ≥70). These trends are largely consistent with prior studies of age trends in disease^26–29^. Meanwhile, some cluster groups demonstrated strong sex influences on comorbidity patterns. Clusters 62 and 2 (96.2% and 91.4% female, respectively) are enriched in genitourinary diseases, and cluster 51 (78.1% female) is enriched in pregnancy complications (Fig. 2d).

Within individual clusters, we observed a consistent scale-free network architecture of phenotype occurrences across all 70 clusters (Spearman’s *ρ* between −0.74 and −0.9, Fig. S2a)^30^. This structure is absent when analyzing the 100,272 participants as a whole group (Fig. S2b). For illustration, cluster 47, enriched in neoplasm (Fig. 2g), with high odds of having bladder, prostate, and kidney cancers, accompanied by metastasis and some circulatory diseases. These top enriched phenotypes also appeared more frequently than non-topic phenotypes (Fig. S2c). These top enriched phenotypes might suggest this cluster contains patients with metastatic prostate cancers and spread to the kidney, as reported by prior studies^31–33^. In addition, some treatments can increase the risk of cardiovascular diseases and are the leading cause of death in men with prostate cancer^34,35^.

### Age and sex are associated with underlying cluster structures and influence the onset time and prevalence of the phenotype

**Fig. 3:**
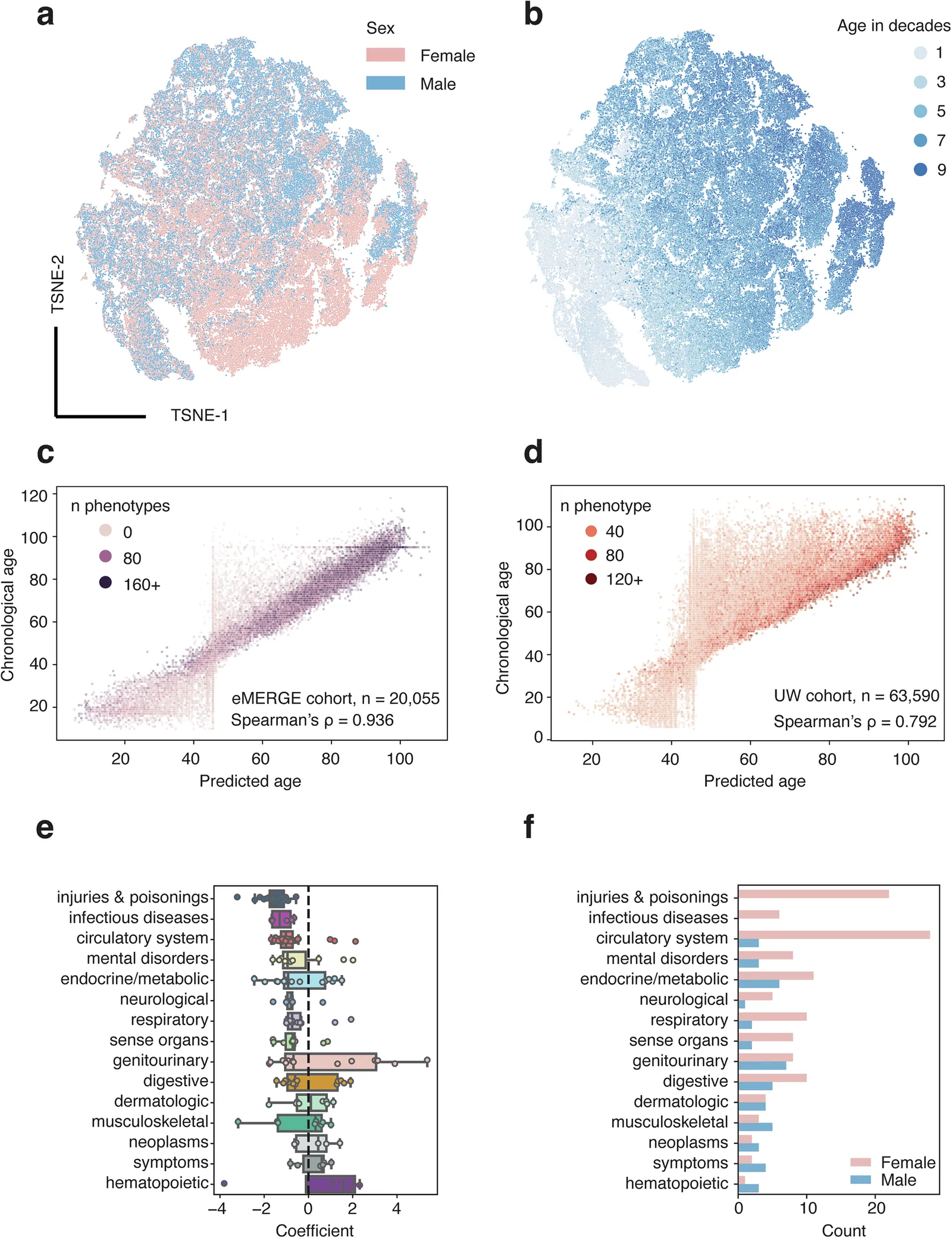
Age and sex are strongly associated with cluster structure and phenotype development. Scatterplot showing T-SNE visualizations of 100,272 patients from the eMERGE cohort colored by sex (**a**) and age (**b**). Scatterplot showing phenotype onset age accurately predicts patient age in **c** the eMERGE cohort and **d** the UW cohort as a validation cohort. The *x*-axis indicates predicted age and the *y*-axis indicates actual age. Points are colored by the number of phenotypes each patient has. The horizontal bands near age 95 arise from the upper-age censoring rule applied at specific recruitment sites. Vertical banding indicates insufficient information, resulting in the model defaulting to the average population age. **e** Boxplot showing the onset time differences of 206 phenotypes that are significantly different between the two biological sexes after Bonferroni multiple-test correction. A negative coefficient indicates late onset in females, while a positive coefficient indicates late onset in males. **f** Barplot showing the number of late-onset phenotypes grouped by each phenotype category in females (pink) and males (blue).

Although patient clusters are well-separated by phenotype categories (Fig. 2a), TSNE-1 and TSNE-2 revealed striking associations related to age and sex (Fig. 3a, b). Using analysis of variance (ANOVA) including site, genetic ancestry, age, and sex as covariates, we found that variation along TSNE-1 is primarily driven by age (Table 1), whereas TSNE-2 is most strongly associated with sex (Table 2). These findings highlight age and sex as the predominant factors shaping phenotypic patterns and patient similarity. This finding prompted a further investigation into the complex relationships among age, sex, and phenotypes.

**Table 1:** ANOVA table explaining TSNE-1

	Sum of square	Degrees of freedom	F-statistics	*p*-values
sex2	5.43e + 04	1.0	420.13	3.55e-93
site	6.79e + 06	11.0	4776.69	0.0e + 00
genetic_ancestry_race	4.82e + 04	2.0	186.47	1.47e-81
***age***	***3.52e*** + ***06***	***1.0***	***27,216.48***	***0.00e*** + ***00***

**Table 2:** ANOVA table explaining TSNE-2

	Sum of square	Degrees of freedom	F-statistics	*p*-values
***sex***	***2.91e*** + ***06***	***1.0***	***13,360.27***	***0.00e*** + ***00***
Site	3.15e + 06	11.0	1314.73	0.00e + 00
genetic_ancestry_race	3.02e + 05	2.0	694.32	3.39e-300
Age	2.35e + 06	1.0	10,785.82	0.00e + 00

Investigating the connection between age and phenotypes, we found that the relationship between the number of new phenotype gains and patient age follows a linear trend across ages 20 to 70 (Fig. S3a), emphasizing the impact of aging. Using gradient boosting tree models, we found that the presence or absence of phenotypes allowed accurate estimation of chronological age (Fig. S3b, Spearman’s *ρ* = 0.848, absolute mean differences = 7.39). More importantly, incorporating phenotype onset timing drastically improved the performance (Fig. 3c, Spearman’s *ρ* = 0.936, absolute mean differences = 3.52). This performance is surprising and comparable to a few studies that used DNA methylation biomarkers to predict age^36,37^. An external evaluation of the model trained on the eMERGE dataset using UW EHR data without fine-tuning (“Methods”) further confirmed this relationship between phenotype onset time and aging (Spearman’s *ρ* = 0.792, *n* = 63,590, absolute mean differences = 7.54, Fig. 3d). The prediction accuracy itself depends on age and the number of phenotypes an individual has (Fig. S3c–f). Notably, the most critical features in chronological age prediction identified by SHAP are also phenotypes associated with aging, such as essential hypertension, hyperlipidemia, and osteoarthrosis (Fig. S4a–c)^38–42^. This suggests that an individual’s overall health and ageing process is reflected in the timing of phenotype onset, revealing an essential link between healthspan and lifespan.

On the other hand, sex affects the prevalence and also partially shifts the onset time of phenotypes. We observed significant differences in the prevalence of phenotypes (*n* = 1136, “Methods”, Fig. S5a–c) between the two biological sexes, calculated based on the odds of phenotype occurrences. By examining the intersection of statistically significant results in the odds of phenotype prevalence and phenotypes significantly correlated with TSNE-2 (“Methods”), we identified 946 phenotypes (out of 1857) that exhibit different prevalence between males and females with high confidence (Fig. S5a–c). Furthermore, we also found that the onset age differs considerably between males and females in many phenotypes. Predominantly, females have an earlier onset of phenotypes across almost every category (Fig. S5d). This initial result contradicts a study from Denmark, where females are usually diagnosed later than males^43^. With a further investigation of potential biases, we noticed that only 48.2% of their participants are female, while our cohort (the eMERGE Network) is 54.1% female (Table 3). Notably, we also observed a significantly greater average number of visits in females (mean 99.68, Table 3) than in males (mean 86, Table 3, *p* = 4.72 × 10^−68^) in our cohort. Correspondingly, females also had a significantly younger age at first hospital visits than males (*p* = 2.70 × 10^−50^, Table 3). These results suggest that earlier age of diagnosis among females might reflect a type of surveillance bias by which females had more opportunities to receive diagnoses^44–47^. These factors can also contribute to a higher baseline chance of a female getting diagnosed early After controlling for the number of hospital visits and age at first hospital visit and adjusting for multiple testing correction, only 204 phenotypes remained statistically significant, with 140 showing later onset in females and 64 in males (Fig. 3e, f), consistent with the previous study showing that females are diagnosed later than males^43^.

**Table 3:** Demographics of sex differences in the eMERGE cohort

	Male	Female	Test for differences
Number of participants	46,051 (45.9%)	54,221 (54.1%)	Chi-square test *P* = 8.70e-147
Mean visits (mean and standard deviation)	86.05 ± 130.27	99.68 ± 145.72	Student’s *t*-test *p* = 4.72e-68
Median visits (median and interquartile)	29.0 (9, 112)	34.0 (10, 137)	Wilcoxon rank-sum test *p* = 1.59e-56
Age at first hospital visits (mean and standard deviation)	42.04 ± 23.38	41.02 ± 21.08	Student’s *t*-test *p* = 4.36e-12
Age at first hospital visits (median and interquartile)	48.0 (23.82, 60.0)	43.06 (25.94, 56.95)	Wilcoxon rank-sum test *p* = 4.56e-47

### Crucial but receding role of genetics in phenotype development during the aging process

**Fig. 4:**
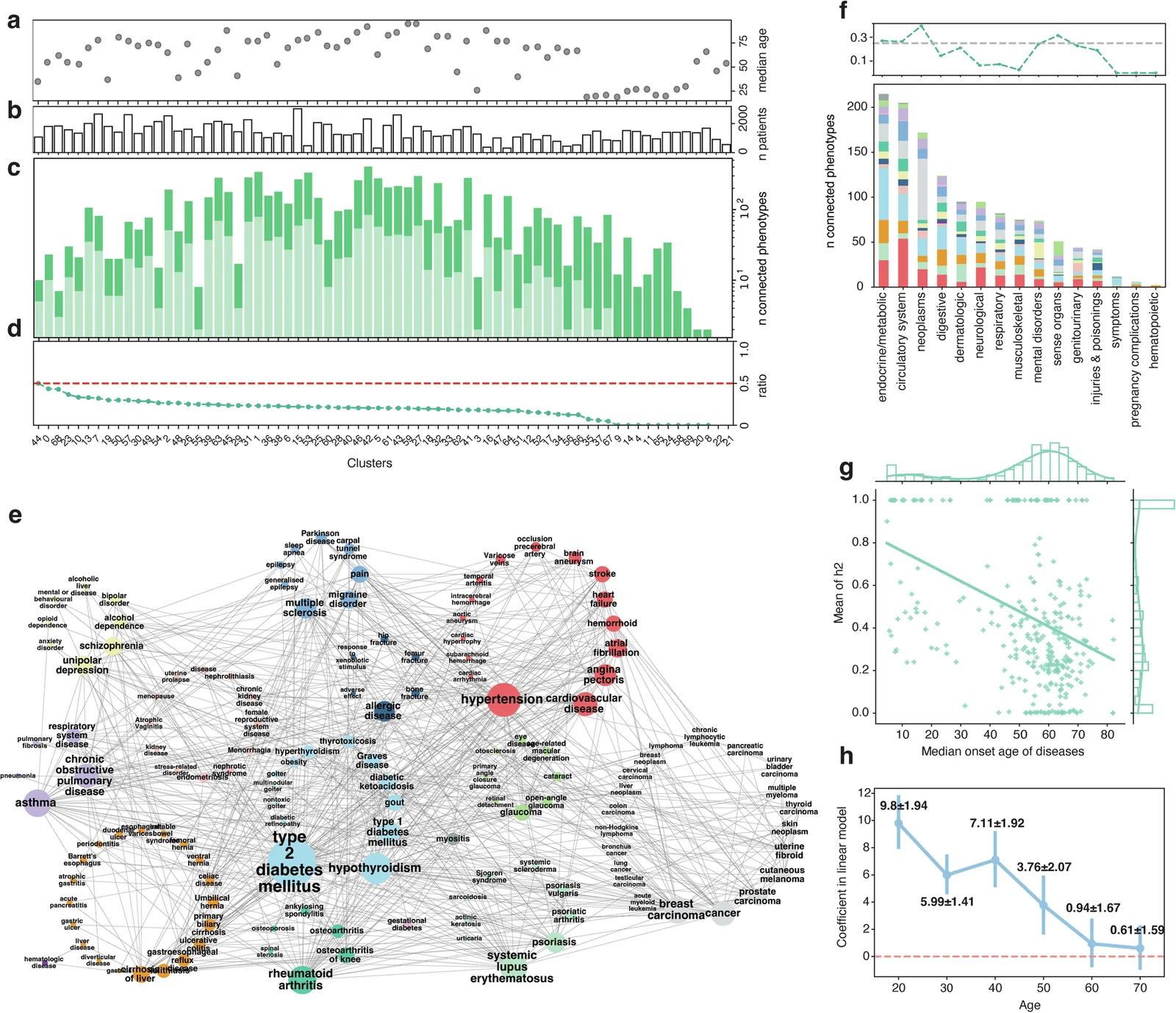
Genetics and the interplay between aging in phenotypes. **a**–**d** Number of observed pleiotropies (defined by the number of significantly enriched phenotypes in a cluster that have shared genetic components) across clusters. **a** The median age of clusters; **b** represents the number of patients in the corresponding cluster; and **c** indicates the number of phenotypes that have shared genetic components (light green) and the number of tested phenotypes (dark green); **d** represents the proportion of connected phenotype (cross-phenotype association). **e** Network plot of genetically connected phenotypes grouped and colored by phenotype groups. Each edge represents a connection and each node represents a unique phenotype. The node size is scaled according to the number of connections the phenotype has. **f** Barplot (bottom) showing the number of genetically connected phenotypes by phenotype groups (*x*-axis). The color of the bars indicates the phenotype group, which is used to evaluate intra- and inter-group cross-phenotype association. The top line plot indicates the percentage of intra-group cross-phenotype association (a phenotype connected to another phenotype in the same group). **g** Scatterplot showing the relationship between heritability (h2) and median onset age of phenotypes (Spearman *ρ* = -0.37, *p* = 4.87e−12). **h** Pointplot showing the coefficient of genetic distance (the *y*-axis) in the phenotype distance regression model stratified by different age groups (the *x*-axis), with the vertical bar indicating 95% confidence interval.

Several studies have quantified and examined the role of pleiotropy in phenotype development; however, its prevalence and contribution to phenotype development at the population level remain poorly understood^48,49^. Using genetic association data from the GWAS catalog^50^, we assessed the prevalence of cross-phenotype associations—a proxy of statistical pleiotropy by quantifying significantly enriched phenotypes within each cluster that shared at least one common SNP. Our findings indicate that cross-phenotype associations are present in most clusters, with cluster 44 exhibiting a notably high level —50% of the phenotypes evaluated in this cluster have shared genetic components (Fig. 4a–d). Statistically, we found that in 57 out of 70 clusters, the enriched phenotypes were significantly more likely to share at least one common SNP, after applying Bonferroni correction for multiple testing (Supplementary Table 1). This finding suggests that shared genetic components contribute significantly to the formation of patient clusters and similar comorbidity patterns. When summarizing all the genetically connected phenotypes, we found a few phenotypes shared abundant connections with others, including type 2 diabetes, hypertension, cardiovascular disease, and hypothyroidism (Fig. 4e). The fact that most connected phenotypes showed cross-category connections suggests complex biological processes governing multi-tissue interactions (Fig. 4f). Among all categories, endocrine/metabolic phenotypes showed the most connections, with the least being hematopoietic. One interesting finding is that neoplasms share the most intra-categorical connections, with about 50% of connected phenotypes also from the neoplasm group, consistent with the known effect of cancer-pleiotropy^51–53^.

As age and sex both contribute to cluster membership, phenotype prevalence, and onset timing, we decided to jointly scrutinize the contributions of age, sex, and genetics in human phenotypes. We derived the first two principal components (PCs), which together explained 63.8% of the variance, from the numerical patient embeddings and treated them as digital phenotypes. We then performed regression analyzes to assess the contributions of age, sex, genetic PCs, and study sites (see “Methods”). While all variables showed significant associations with the first two PCs, age emerged as the strongest determinant (Fig. S6, and Tables 4 and 5). Taking a complementary approach, we paired random patients, quantified the phenotype distances using the embeddings, and employed a linear model to evaluate the contributions of age gap, sex differences, sites, and genetic distances to their phenotype distances (below referred to as phenotype distance regression analysis, see “Methods”). Still, this analysis yielded consistent results showing that all variables played crucial roles in determining the phenotype distances (Fig. S6 and Tables 4 and 5), with age consistently being the most critical contributor (Fig. S6 and Tables 4 and 5), followed by sex and sites, then genetic distances.

**Table 4:** ANOVA table on digital phenotype PC1

	df	sum_sq	mean_sq	F	PR( > F)
***age***	1.0	26,468.703023	26,468.703023	***64,426.157262***	0.000000e + 00
site	11.0	97,356.231831	8850.566530	21,542.725030	0.000000e + 00
sex2	1.0	1315.475202	1315.475202	3201.932947	0.000000e + 00
Genetic PC1	1.0	97.750641	97.750641	237.929987	1.281736e-53
Genetic PC2	1.0	0.083436	0.083436	0.203087	6.522414e-01
Genetic PC3	1.0	11.999929	11.999929	29.208431	6.514354e-08

**Table 5:** ANOVA table on digital phenotype PC2

	df	sum_sq	mean_sq	F	PR( > F)
***sex***	1.0	10,565.402049	10,565.402049	***21,217.331195***	0.000000e + 00
age	1.0	1337.194577	1337.194577	2685.340329	0.000000e + 00
site	11.0	7304.166336	664.015121	1333.468304	0.000000e + 00
Genetic PC4	1.0	171.853656	171.853656	345.114735	6.613682e-77
Genetic PC1	1.0	145.110161	145.110161	291.408726	3.034129e-65
Genetic PC3	1.0	64.052130	64.052130	128.628826	8.525990e-30

Interestingly, Jia et al. found that the heritability of phenotypes is significantly negatively correlated with the onset age^54^. To further examine the role of genetic factors in phenotype onset across age groups, we obtained heritability estimates from Jia et al. and mapped them to 285 phenotype terms in the eMERGE cohort (see “Methods”). Consistent with the findings of Jia et al., we observed a significant inverse correlation between h2 and median onset age (Spearman *ρ* = −0.37, *p* = 4.87 × 10^−12^, Fig. 4g). From another perspective, we split patients into different age groups and re-performed the phenotype distance regression analysis again (see “Methods”). This time, we found a progressive decline in the genetic contribution to phenotype distances with increasing age, most notably among individuals in their 60s and 70s, where the confidence intervals overlapped with zero (Fig. 4h), indicating no significant genetic effect. Collectively, these findings highlight a substantial yet gradually diminishing role of genetic factors in phenotype development with aging, consistent with the observed reduction in heritability for late-onset phenotypes.

### Clusters disparity in CCI index and mortality reveals the link between premature aging and phenotypes onset time

**Fig. 5:**
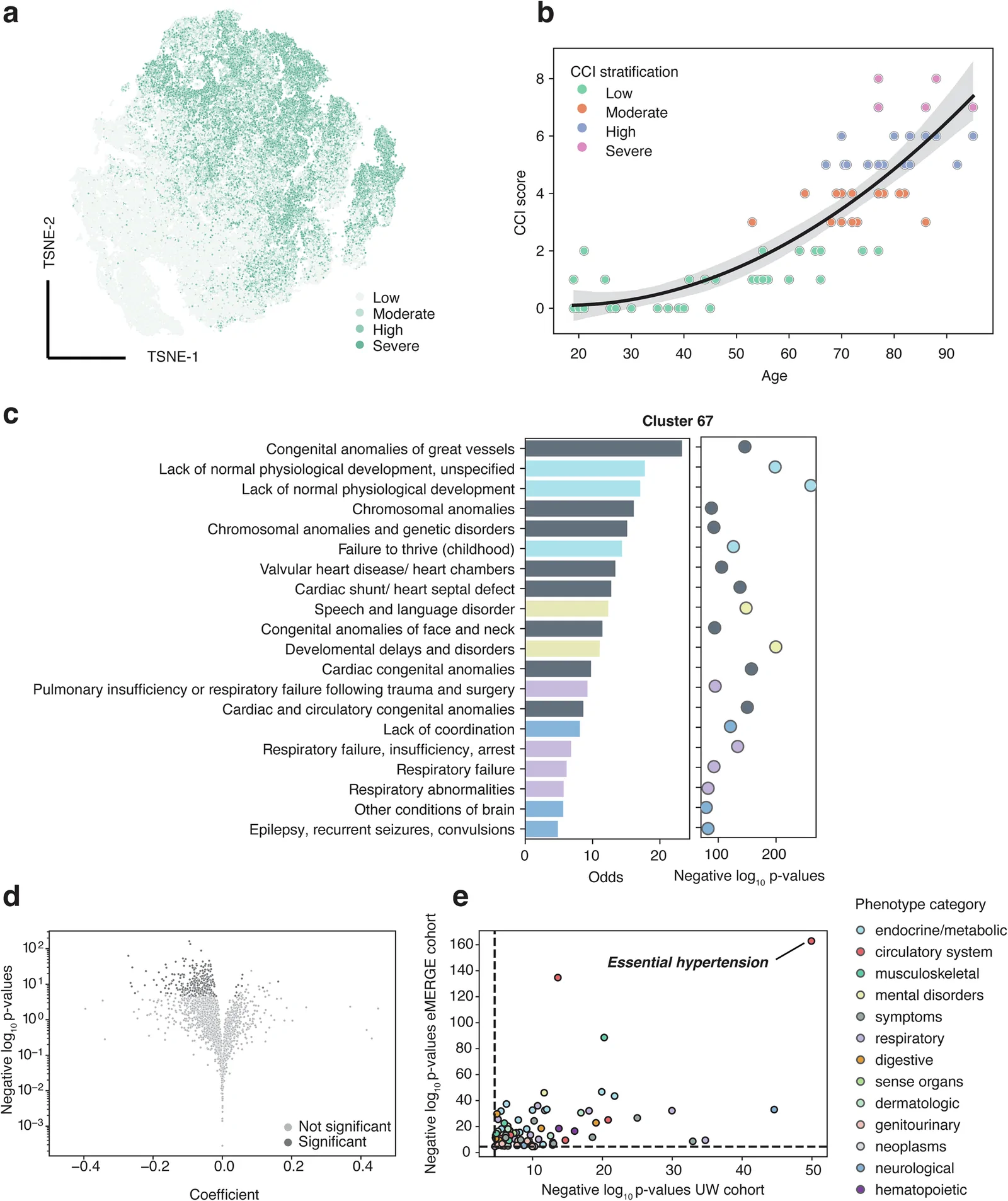
Cluster disparities in CCI index and mortality reveal premature aging phenotypes. **a** Scatterplot showing T-SNE visualizations of 100,272 patients from the eMERGE cohort colored CCI index. **b** Scatterplot showing the median CCI index of each cluster group (the *y*-axis), regression against age (the *x*-axis). Points above the regression line indicate high-risk groups for the corresponding age. **c** Barplot showing the top 20 enriched phenotypes in cluster 67 (left) and corresponding negative log10 *p*-values. Cluster 67 is a cluster group of young patients (median age 21) with relatively high risks (median CCI index = 2). Points and bars are colored according to the phenotype group. **d** Scatterplot showing the onset age of 327 phenotypes significantly (black) associated with the CCI index. A negative coefficient (the *x*-axis) indicates the early onset of these phenotypes associated with higher CCI. **e** Scatterplot showing 82 phenotypes that have their age significantly associated with CCI in the eMERGE cohort (negative log_10_ *p*-values shown in the *y*-axis) and mortality in the UW cohort (negative log_10_ *p*-values shown in the *x*-axis).

To understand if clusters exhibit discrepancies in overall health, we examined the Charlson Comorbidity Index (CCI)—a proxy for 10-year mortality risk (“Methods”)—across cluster groups. While CCI is generally correlated with age, we observed clear stratifications of CCI scores among clusters within defined age ranges (Fig. 5a, b). As a predictor of mortality, a CCI score of 2 corresponds to approximately a 90% 10-year survival probability, while a score of 4 corresponds to about 53%^55–58^. To further characterize comorbidity burden among higher-risk patients, we focused on clusters with moderate to high CCI scores (≥2) within certain age bins (Fig. S7). Among these clusters, we noticed a young cluster group (cluster 67, median age = 21, Figs. 5c and S7) with moderate median CCI (median CCI = 2), with over 20% having chronic obstructive pulmonary disease (COPD), and about 20% with congestive heart failure, cerebrovascular disease (CEVD), and cancer (CANC). This finding is crucial in characterizing high-risk comorbidities in teenagers and enables potential early intervention for these young participants. Other clusters exhibit aging-related health issues, especially chronic disorders, such as cluster 38 (median age 53) and Cluster 5 (median age 63), two similar clusters with over 40% of COPD and mild liver disease (MLD), and also high proportions of diabetes without complications (DIAB, over 30% in Cluster 38 and over 50% in Cluster 5). Cluster 66 (median age 67) and cluster 34 (median age 70) have high proportions of CANC and MLD, with cluster 66 having a higher (over 40%) rate of renal disease (REND). All other older clusters, with a median age greater than 70 (clusters 53, 1, and 64), shared similar patterns in CCI comorbidities, except that cluster 52 had a high proportion (over 80%) of REND. To confirm that these clusters exhibit higher mortality, we randomly selected approximately 100k patients (the same size as the eMERGE cohort) from the University of Washington (UW) EHR cohort with mortality data as an external evaluation (“Methods”). We applied the GMM trained on the eMERGE cohort to the UW data and assessed whether patients assigned to the high-CCI clusters identified in eMERGE (clusters 67, 38, 5, 66, 34, 28, 52, 1, and 64) exhibited an elevated mortality risk. Consistent with our findings in the training cohort, patients in these high-risk clusters have significantly higher mortality rates compared to those in other clusters (odds ratio = 4.21; *p* = 4.02 × 10^−152^; Fig. S8a–c). The result remains unchanged after adjusting for sex and age in a linear model predicting mortality (odds ratio = 2.65, *p* = 1.32 × 10^−82^, “Methods”). At the individual cluster level, 7 out of 9 clusters demonstrated significantly increased mortality (Fig. S8c), with the two non-significant clusters (52 and 64) limited by small sample sizes (*n* = 22 and *n* = 2, respectively). Together, this analysis explored the feasibility of quantifying patient risk using comorbidity patterns in a data-driven manner and motivated further investigation into the underlying mechanisms of high-risk comorbidity profiles.

To understand the mechanism underlying the link between phenotypes and increased mortality rate in these clusters, for each phenotype (*n* = 1857, defined by phecode), we systematically used the onset age of each phenotype as an independent instrument, fitting a linear model including age, sex, phenotype onset time, and an interaction term between age and phenotype onset time to predict the CCI (“Methods”). We identified 327 phenotypes whose onset age significantly influenced CCI (Fig. 5d). The strongest association was observed for essential hypertension (phecode 401.1, *p* = 1.31 × 10^−163^, coefficient = −0.095; Figs. 5d, and S9a), where the negative coefficient indicates that earlier onset is linked to higher comorbidity burden. Consistent with our findings, other studies also reported that early onset of hypertension can lead to premature death^59,60^. Notably, 317 out of the 327 significant phenotypes exhibited negative coefficients, suggesting that earlier onset of most conditions is constantly associated with increased CCI, and thus, shorter healthspan and lifespan. We externally validated the relationship between onset age and mortality using a random selection of 100K participants from the UW cohort, fitting Cox proportional models with each phenotype onset age as an instrument to predict overall mortality, controlling for age and sex (“Method”). The onset age of essential hypertension again ranked as the most significant predictor of overall mortality (Fig. S9a). Five phenotypes lie within the intersection of the top 20 significant phenotypes in both cohorts: essential hypertension, joint pain, type 2 diabetes, hyperlipidemia, and chronic pain (Fig. S9a, b). In total, 82 phenotypes are significant in both cohorts (Fig. 5e). As shown previously, phenotype onset age is crucial for predicting chronological age (Fig. 3c,d); the fact that onset age of phenotypes can affect overall mortality indicates links between certain phenotypes’ onset and premature aging, such as essential hypertension^61–63^. Together, these findings highlight a connection between phenotype onset age and overall health, prompting further investigation into how variation in the aging process relates to mortality.

### Survival selection and characteristics of successfully aged participants

**Fig. 6:**
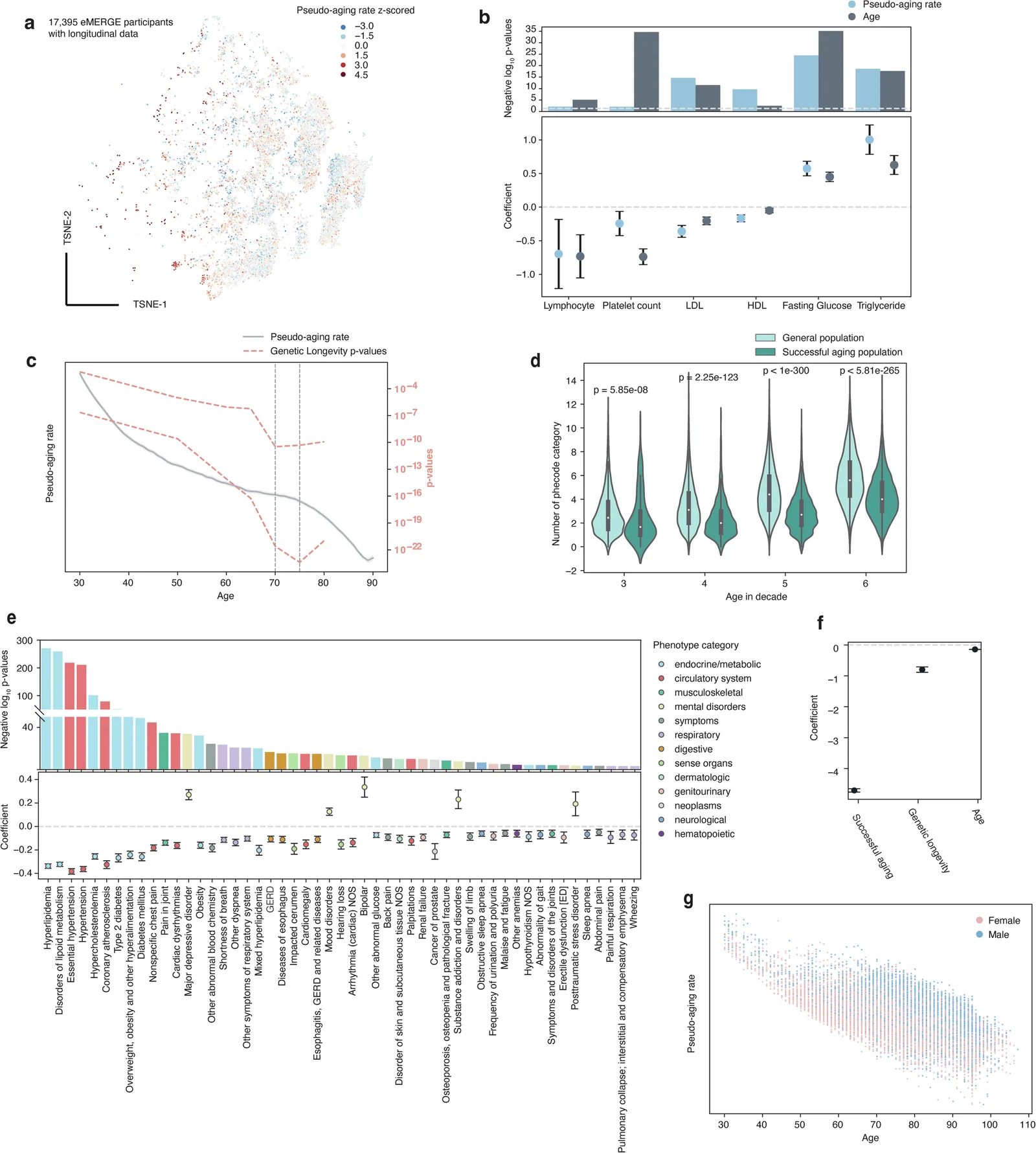
Survival selection and characteristics of successfully aged participants. **a** Scatterplot showing T-SNE visualizations of 17,395 patients from the eMERGE cohort with longitudinal data colored by z-scored aging rate. **b** The aging rate predicts labs that are known to be associated with aging. The bottom is a point plot with a confidence interval showing the coefficient of age rate and age in linear models adjusted for sex, sites, and genetic ancestry, predicting each lab respectively in the corresponding *x*-axis. The top bar plot represents negative log_10_ *p*-values of each lab. **c** A line plot illustrating how the pseudo-aging rate and genetic longevity jointly revealed survival selection in patients aged over 75. The grey line represents the average aging rate across 17,395 patients in the eMERGE cohort with longitudinal data. For each point along the *x*-axis, the two pink dotted lines display *p*-values comparing genetic longevity PRS for individuals older than the given age versus those of the same age or younger. The upper pink dotted line corresponds to the subset with longitudinal data, while the lower line reflects the full cohort of 100,272 eMERGE patients. For each point along the *x*-axis, *p*-values compare individuals older than the given age with those of the same age or younger. **d** Successfully aged participants (aged ≥ 75) have less multi-group comorbidity. The violin plot shows the number of phenotype categories (the *y*-axis) individuals have at a specific age group in decades (the *x*-axis). Darker green colors represent successfully aged participants, and lighter colors represent others. **e** 51 out of the 82 phenotypes shown in Fig. 5e have a significant association between aging rate and onset time. Each dot (bottom) here represents a phenotype (the *x*-axis) and its coefficient (the *y*-axis) in the linear model predicting onset time. A negative coefficient indicates an increased aging rate associated with an early onset time. A positive coefficient indicates an increased aging rate associated with a later onset time. The bar plot (top) represents negative log_10_ *p*-values. **f** Stripplot showing that the aging rate is drastically different at baseline between the successfully aged population and others while mildly associated with aging. **g** Scatterplot showing the pseudo-aging rate (the *y*-axis) between two biological sexes across different ages (the *x*-axis).

To understand the link between aging and phenotype development, we examined the aging process by quantifying pseudo-aging rates in 17,395 eMERGE participants with longitudinal data spanning 20 years. (Fig. 6a, “Methods”). Specifically, we implemented a gradient boosting regression model using embedding features to predict patient age. We applied a random sampling method during training to ensure a uniform age distribution to avoid overfitting to specific age groups (Fig. S10a). We then quantified the difference between the model-predicted and actual age and defined it as pseudo-aging rate (“Methods”, Fig. S10b). The pseudo-aging rates were also significantly associated with longitudinal changes in various laboratory tests that are known to be associated with aging, including platelet counts, lymphocyte counts, fasting glucose, triglycerides, LDL, and HDL (“Methods”, Fig. 6b), consistent with previous findings^64–71^.

Interestingly, we observed a deceleration in pseudo-aging rates with increasing age among the 17,395 participants with longitudinal data, particularly after age 75 (Fig. 6c). This indicates that younger individuals are predicted as older, and older individuals as younger (Fig. S10c, d). We examined other observational studies and observed a similar pattern^36,72^. We hypothesize that these trends suggest underlying heterogeneity in the population structure. Upon closer examination, we interpret this phenomenon as a survival selection common in observational studies: individuals over 75 are likely to represent a selectively healthier, longer-lived subgroup, while younger and middle-aged individuals reflect a broader, more general population before selection. To validate this, we focus on investigating the marked slowdown in the age group 60 to 75 (Fig. 6c). We compared genetic longevity polygenic risk scores (PRS) between patients younger and older than specific age thresholds (“Methods”). The strongest stratification point was age 70 among the 17,395 longitudinal participants (*p* = 3.35 × 10^−11^), and age 75 in the full cohort of 100,272 individuals with genetic data (*p* = 4.06 × 10^−24^; Fig. 6c). These two results matched the aging rate turning point (Fig. 6c), further suggesting the discrepancies between these two populations. We also performed a simple simulation experiment modifying the population structure in different age groups and identified the same trend (“Method”, Fig. S10e, f).

Building on our findings and existing life expectancy data, we defined “successfully aged” participants as those aged ≥75 and identified several distinguishing characteristics^73–75^. As aging is proven to be multi-system^72,76,77^, we examined and found that successfully aged participants have a lower cross-category disease burden since early life (Fig. 6d). Reasonably, they also showed a delayed onset in most mortality-associated phenotypes (51 out of 82) we identified in the previous session, except for five mental disorders (Fig. 6e, “Method”). Using linear regression, we found that the aging speed of successfully aged participants has a much lower baseline (Fig. 6f), and the influence of participant age is much smaller. Although successfully aged patients are more likely to have a longer follow-up, potentially introducing biases, we found that after adjusting for the number of years of follow-up, the above analysis remains significant and solid (Fig. S11). Interestingly, females also showed a consistently lower pseudo-aging rate than males across all age groups (Fig. 6g), which can help explain their overall better life expectancy than males.

## Discussion

While machine learning and deep learning have shown remarkable success in large-scale data analysis, uncovering clinically meaningful patterns from EHR data still requires more nuanced and targeted data mining approaches. In this study, we applied an unsupervised learning method to patient-level EHR data and identified distinct clusters, each characterized by a unique pattern of enriched phenotypes. Still, age and sex emerged as dominant factors shaping the phenotype prevalence and onset. Certain patterns of phenotype co-occurrence were strongly supported by cross-phenotype associations analysis. A careful characterization of overall mortality in each cluster identified phenotypes’ onset associated with elevated mortality, revealing links between premature aging and the early onset of some phenotypes.

Phenotypes were often encoded as binary variables (presence vs absence) and were studied without examination of comorbidity^78^. In our analyzes, we represent patients’ phenotypes as numerical embeddings trained from our previous work (referred to as digital phenotype), and assess the contributions of genetics, age, and sex to phenotype development. We identified the critical role of genetics in explaining phenotype discrepancy, together with age, sex, and sites as covariates. Notably, many of the genetically linked phenotypes belong to different disease categories (Fig. 4e), serving as observational evidence to further study the complex biological processes that may act across organ systems in triggering the development of additional comorbid conditions. Yet among all variables, age consistently appeared to be the most crucial contributor to phenotype differences. Moreover, the genetic contribution recedes when an individual enters advanced aging states, consistent with previous findings of decreased heritability in late-onset diseases. These findings suggest aging as a universal determinant of phenotype onset timing.

This study confirms that age and sex are dominant factors in influencing phenotype prevalence and onset patterns. A key finding is that the onset age of specific phenotypes can reliably predict participants’ chronological age, with the top predictive features also strongly associated with CCI and mortality risk, such as essential hypertension. Prior studies also reported the major role of onset age and sex in the onset and outcomes of certain phenotypes^79–82^. Our analysis also revealed a connection between phenotype onset time and overall mortality. Specifically, we identified 82 phenotypes whose onset age was significantly associated with overall mortality in two independent cohorts. These results suggest that the earlier onset of these phenotypes reflects premature aging and is associated with an elevated mortality risk. Several studies explored and discussed the underlying mechanisms of premature aging and disease onset, including increased liability, different causes, genetics, and molecular-level changes^83–85^. Yet the field is still new, and the underlying mechanisms remain poorly understood.

Importantly, the survival selection we identified in our cohort likely reflects a broader issue common to observational studies, where older individuals represent a selected group of survivors who have successfully aged. Characterization of this successfully aged population revealed several distinguishing features, including lower cross-tissue disease burden, a stable and slower intrinsic aging rate, and delayed onset of mortality-associated phenotypes. These findings are consistent with prior studies reporting a generally lower disease burden and a delayed onset of diseases^86,87^ in successfully aged populations. The biological aging process remains an unresolved puzzle, exerting a critical influence on an individual’s health status and lifespan. Our results extend this understanding by showing that estimated aging rate and genetic longevity can clearly distinguish patients with survival advantages from the general population. This raises the question: if aging is not genetically programmed, to what extent—and through which factors—can healthspan be extended^88^?

Several prior EHR clustering studies focused on specific disease conditions, predefined phenotypes, disease subtypes^9,10,89^; or trained unsupervised models primarily for downstream supervised prediction tasks^90,91^. Here, we applied an unsupervised learning method to perform phenome-wide stratification and characterization of large-scale patient-level EHR data with minimum annotation, uncovering strong interactions among phenotypes, age, sex, and genetics. Our findings have implications for clinical risk stratification, predictive modeling, and understanding disease mechanisms in aging. Specifically, clustering patients by comorbidity patterns revealed high-risk groups and novel clinical associations, while incorporating phenotype onset age into risk models improved predictive accuracy and highlighted its potential as a proxy for biological aging. We also identified survival selection, underscoring the importance of accounting for population heterogeneity in observational research. For future research, there is a need for novel or more effective patient embeddings to uncover complex trends and identify unknown patterns and mechanisms through stratification analysis. Experiments with specific downstream optimization can tune the embedding models to capture variations among patients in high-dimensional data. In terms of outcome analysis, future research can focus on more granular health outcome evaluations, which can include examining laboratory results, medication usage, and observational data. A more refined analysis of data, incorporating multi-modal sources and complex health metrics, can enhance feature analysis for patient stratification and improve our understanding of disease etiology.

This work has a few limitations. First, though the eMERGE EHR data is a thorough and diverse source, it is a research cohort that might not be a suitable representation of the general population. Second, the data is a cohort prioritized for genomic studies that lacks appropriate health outcomes, such as length of stay, annotations, healthcare utilization, socioeconomic status, environmental factors. This makes certain analysis less adjusted and the limited more meaningful downstream analyzes. For example, the data lacks more granular data, such as labs, medications, and observations, which could help provide more detailed patient stratification, even deep phenotyping. Third, the patient embedding vector lacks meaningful information of clinical progression annotation, making it undesirable for progressive diseases or episodic conditions. Fourth, misdiagnosis can also harm the general embedding qualities, as many have discussed and identified evidence of misdiagnosis in the EHR system, especially for complex phenotypes like psychiatric diseases^92–94^.

In summary, we introduced a novel unsupervised learning approach to characterize patient-level EHR data, enabling the identification of distinct comorbidity patterns and revealing key relationships among age, sex, genetics, and phenotypes. By linking these patterns to overall mortality—a critical health outcome—we uncovered a previously underexplored connection between phenotype onset and the aging process. Using a longevity PRS, we further stratified the older population and provided evidence of survival selection in observational studies. Our analyzes validated the critical link between the aging process, health status, and mortality, and demonstrated that the phenotype onset time serves as a robust indicator of these states. Collectively, this work showcases the potential of unsupervised EHR analysis for uncovering clinically and biologically meaningful patterns in real-world data.

## Methods

### Data and cohort description

#### The eMERGE cohort

The eMERGE cohort comprises data from three phases (I–III) of the eMERGE Network, encompassing approximately 100,272 participants from 12 diverse clinical sites^25^. This dataset includes basic demographics (age, sex, genetic race, clinical sites), diagnoses (ICD-9 and ICD-10), procedures (CPT4), and select laboratory results. The diagnosis codes were mapped to phecodes and used to define patients’ phenotypes^95^. The dataset is accessible upon request and discussion.

#### The UW EHR cohort

The UW EHR cohort (*n* = 840,000) was introduced in our previous work^10^. The UW EHR cohort was mainly used for validation purposes. This dataset included basic demographics (age, sex, self-reported race), diagnosis (ICD-9 and ICD-10), procedures (CPT4), and mortality status. In this work, we randomly queried 100,000 participants from the UW EHR cohort, which is the same size as the eMERGE Network (training data), serving as a validation cohort for high-risk group analysis and mortality analysis. Demographic data of both cohorts can be found in the Supplementary Table 1 from our previous work^10^.

### IRB and ethical declaration

This study was conducted in accordance with the Declaration of Helsinki and institutional ethical standards under the IRB protocol STUDY00015886: Generalizability Assessment of the eMERGE study on the University of Washington Medical Center Population. All data extracted from the University of Washington were de-identified, covered under this IRB approval, and stored on a HIPAA-compliant server. No human subject was involved in the study.

### Unsupervised learning for patient embedding

The numerical patient embeddings used for patient clustering analysis were generated from our previous work^10^. For the main analyzes, we collected all longitudinal embedding events for each patient and averaged across all years to generate patient embeddings. For the aging and survival selection analysis, we used the longitudinal patient embedding of patients who have at least 20 years of follow-up (*n* = 17,395) to model the pseudo-aging rate. During the modeling phase, predicting current patient age, we only included time points up to the longitudinal event for patient embeddings to avoid leaking information about future longitudinal events.

### Clustering performance comparison between GMM and K-means

We included all 50 numerical embedding features and all 100,272 eMERGE participants in the clustering analysis, which is a matrix of 100,272 × 50. We used the GMM and K-means from Scikit-learn^96^ version 0.24.2. We first found that the number of topics identified is highly similar between K-means and GMM (Fig. S12a, Spearman’s *ρ* = 0.907). However, when comparing the number of significantly enriched codes in each cluster after the Bonferroni correction, GMM is superior, yielding 52 clusters with higher numbers of significantly enriched codes (chi-square test 16.51, *p* = 4.83e-05). In detail, we sorted the significantly enriched codes from high to low in all 70 clusters for K-means and GMM, respectively. Then we paired these sorted two arrays and found that GMM constantly surpasses K-means (Fig. S12b), with 52 clusters having higher numbers of significantly enriched codes. The chi-square test is performed by comparing the 52 observed higher numbers to an expected 35 numbers if the two methods are equal (in total 70 clusters). More enriched codes in clusters indicate a better and meaningful separation. K-means assumes spherical clusters with equal variance, whereas GMM allows clusters with different covariance structures. Examination of the feature distributions in our dataset indicates patterns consistent with mixtures of Gaussian distributions rather than spherical clusters (Fig. S13). It is therefore reasonable to observe a better performance using GMM. To further quantify the distinctiveness of clusters, we performed pairwise cluster comparisons using Jaccard similarity of enriched phenotypes (Fig. S12d). Specifically, we calculated the overlap between the top 20 enriched phenotypes of each cluster pair (Fig. S12d). This analysis revealed that clusters generated by K-means showed substantially higher overlap (mean 0.16, median 0.125), indicating that many clusters share similar phenotype compositions and may therefore contain redundant information. In contrast, GMM clusters exhibited minimal overlap (mean 0.03, median 0.0), suggesting that GMM identifies more distinct and phenotype-specific patient groups.

### Selection of cluster numbers based on clustering stability, validity, and clinical interpretability

We first assessed various metrics to decide the optimal cluster numbers, including Bayesian information criterion (BIC), Silhouette score, Adjusted Rand Index (ARI), and AMI. Silhouette score inherently favors convex, spherical clusters and therefore tends to perform better for K-means clustering. In addition, it is known to perform poorly in complex high-dimensional datasets and when cluster sizes are highly imbalanced^97–100^. ARI is a pair-counting measure that evaluates whether pairs of samples are assigned to the same or different clusters across clustering solutions. Because pair counts scale quadratically with cluster size, ARI tends to perform best when clusters are relatively balanced and can strongly penalize cluster splits or merges^99,101^. For these reasons, Silhouette score and ARI are not ideal metrics for evaluating clustering performance in our dataset (Fig. S1a, b). In contrast, AMI is based on information theory and is less dominated by large clusters, making it more appropriate when cluster sizes are imbalanced or when small clusters are present^99,101^. As described above, cluster sizes in our analysis range from a few hundred to approximately three thousand patients. Increasing the number of clusters leads to finer granularity in disease groupings (e.g., a single broad cancer cluster separating into multiple cancer-type subclusters), which improves clinical interpretability but is highly penalized by ARI (Fig. S1b). Because cluster sizes are heterogeneous and cluster structure becomes more refined as cluster number increases, we primarily evaluated clustering stability using AMI across bootstrap resampling (Fig. S1c). Across clustering solutions with 15, 30, 50, and 70 clusters, K-means consistently showed higher AMI values than GMM, indicating greater stability under resampling, although both decrease with increased cluster numbers (Fig. S1c). Nevertheless, both approaches produced reasonably high AMI scores overall (median AMI for GMM = 0.68, for K-means = 0.78, both at 70 clusters), suggesting stable cluster structures. Based on these metrics, including BIC for GMM (Fig. S1d), clustering solutions with 15 or 30 clusters may appear optimal. However, these solutions substantially limit clinical interpretability. For example, in the 15-cluster solution, all neoplasm-related cases are grouped into a single cluster containing 9543 patients. This prevents the identification of clinically meaningful subgroups corresponding to different cancer types and instead produces only coarse groupings based on broad disease categories. In contrast, the 70-cluster solution resolves seven distinct cancer-related patient groups, each enriched for different cancer types (https://github.com/suxian06/patient-topic-modeling/blob/main/Topics%20of%20all%2070%20clusters.ipynb). Thus, cluster number cannot be determined solely by maximizing a stability metric. From a clinical perspective, the 70-cluster solution provides substantially more refined and interpretable patient subgroups. Notably, this increase in resolution results in only a minor decrease in stability (AMI 0.74 to 0.68), indicating that cluster structure remains robust.

### Topic identification and enrichment analysis for clusters

We first define phenotype by mapping ICD-9 and ICD-10 diagnosis codes to phecodes. Each phecode represents a unique phenotype (e.g., essential hypertension corresponds to phecode 401.1). For each defined patient cluster, we used Fisher’s exact test (scipy package^102^ version 1.7.1) to compute their phenotype enrichment and used as a descriptive summary of phenotype concentration within clusters. The enrichment comparison was a one-versus-the-rest strategy, meaning each cluster was compared to all the rest of the cohort. This method can capture the relative enrichment of phenotypes while being robust against some high-frequency but tangential phenotypes (such as hypertension in elderly populations). Then, the top 20 enriched phenotypes were selected as a representation. We then mapped these top 20 phenotypes to their phenotype categories (e.g., essential hypertension is mapped to the circulatory system). We defined the most frequent category as the topic for each cluster. The visualization of all 70 clusters is available here: https://github.com/suxian06/patient-topic-modeling/blob/main/Topics%20of%20all%2070%20clusters.ipynb.

### Topological free network of individual clusters

The power law graph representing the topological free disease network is constructed by quantifying the number of times the phenotypes occurred within a specific cluster (in the *x*-axis) and plotted against the frequency of such occurrence time^103^. Spearman correlation coefficient was reported for each cluster, and we observed a consistently high negative correlation, indicating that the topological free network architecture is consistent for all clusters (Fig. S2a).

### Analysis of variance (ANOVA) for TSNE-1 and TSNE-2

TSNE is computed using the Scikit-learn Python package^96^ (version 0.24.2). ANOVA is used to evaluate the contribution of age and sex in patient stratification (represented by TSNE-1 and TSNE-2). We included sites, genetic ancestry, age, and sex together as covariates in the model to predict TSNE-1 and TSNE-2 (see formula below), separately, where we found that the age of participants majorly explains TSNE-1, while sex is most associated with TSNE-2 (Tables 1 and 5). For each component TSNE_*c*_ (*c* = 1,2), we performed regression analysis using the statsmodels Python package^104^ with the following formula and then performed ANOVA based on the regression result using the statsmodels Python package^104^, while *i* indicate each patient:

$${\mathrm{TSNE}}_{c}={\mathrm{site}}_{i}+\mathrm{genetic}\,\mathrm{ancestry}\,{\mathrm{race}}_{i}+{\mathrm{age}}_{i}+{\mathrm{sex}}_{i}$$

### Regression models predicting chronological age

The chronological age is calculated by subtracting the birth year of patients from the year of study (2024). We employed the gradient boosting regressor from Scikit-learn^96^ (version 0.24.2) to predict chronological age. Initially, we constructed a model using binary features, where a value of 1 indicated the presence of specific phenotypes in patients, and a value of 0 indicated their absence. Subsequently, we developed another model using continuous values to represent the onset age of patients’ phenotypes, with 0 again signifying the absence of any phenotype. In both of the modeling phases, 80% of the samples were used for training, and the rest of 20% were used for evaluation. The same random seed was used to ensure that two models using the same training set are evaluated on the same evaluation set. The objective of this analysis was to demonstrate that incorporating more detailed information, the onset age of specific phenotypes, can significantly enhance model performance.

### Phenotype prevalence differences in sex

We evaluated the disease prevalence differences between the opposite sex using two independent methods. We first computed the odds of observing each phenotype between males and females, referred to as the raw odds method. As we noticed the strong stratification effect of TSNE-2 on biological sex in Fig. 3a, we computed the Spearman correlation (scipy^102^ version 1.7.1) between sex and TSNE-2 and referred to it as the TSNE-2 method in this manuscript.

### Phenotype onset time differences in sex

We first computed the onset differences for each phenotype (defined by phecodes) between the two opposite biological sexes using the Wilcoxon rank-sum test, yielding 596 phenotypes with onset time differences. We then corrected potential confounding variables, including age at the first hospital visit and the total number of visits in a linear model, together with sex, to predict onset time, yielding 327 significant onset differences between males and females. The Wilcoxon rank-sum test is implemented using the scipy package^102^ version 1.7.1 from Python. The linear model is implemented using the Python package statsmodels^104^, version 0.12.2. In detail, for each phenotype *i*, we fit the linear model using the below formula:

$$\begin{array}{l}\mathrm{phenotype}\,\mathrm{on}\,\mathrm{set}\,{\mathrm{age}}_{{\rm{i}}}\\ \,\,\,=\mathrm{age}\,\mathrm{at}\,\mathrm{first}\,\mathrm{hospital}\,{\mathrm{visit}}_{{\rm{i}}}+\mathrm{total}\,{\rm{n}}\,\mathrm{hospital}\,{\mathrm{visit}}_{{\rm{i}}}+{\mathrm{sex}}_{{\rm{i}}}\end{array}$$

### Calculating the Charlson comorbidity index (CCI)

The Charlson comorbidity index (CCI) is calculated using Python package comorbidipy^105^ (https://github.com/vvcb/comorbidipy). The calculation follows the standard definition of the Charlson comorbidity index^58^, and the CCI is then used as a representation of the overall 10-year survival estimation.

### Regression analysis predicting CCI using phenotype onset age

For each phenotype, we performed regression analysis treating CCI as the dependent variable and using the chronological age, onset age of phenotype, and sex as independent variables, including an interaction term between chronological age and onset age of phenotype, to investigate if the onset age of phenotype can predict CCI. The linear model was implemented using Python package statsmodels^104^, version 0.12.2. For each patient *i*, the variable CCI_*i*_ is predicted using the following formula:

$${\mathrm{CCI}}_{i}=\mathrm{chronological}\,{\mathrm{age}}_{\mathrm{i}}\times \mathrm{phenotype}\,\mathrm{on}\,{\mathrm{setage}}_{i}+{\mathrm{sex}}_{\mathrm{i}}$$

The full summary statistics are provided in Supplementary Table 2.

### Cox regression modeling using UW EHR data

As described in the UW EHR cohort section, we randomly selected 100 K samples from the UW cohort with mortality data and performed Cox regression analysis^106^. A demographic comparison between the sampled subset and the full UW EHR cohort is provided in Supplementary Table 3 to assess representativeness. For each phenotype defined by phecodes, we fitted a Cox proportional hazards model to evaluate its association with overall survival, using the age at phenotype onset as the primary predictor and adjusting for age and sex as covariates. The model coefficient and *p*-values are then compared with the linear regression model predicting CCI in the eMERGE cohort as a cross-cohort validation. The top associationsare shown in Fig. S9. The Cox proportional hazard model is implemented using the Python package lifelines^107^ version 0.27.4 with the CoxPHFitter function. The full summary statistics are provided in Supplementary Table 4.

### Principal component analysis of age, sex, sites, and genetics contributing to digital phenotypes

We performed principal component analysis (PCA) on the embeddings of 100,272 patients from the eMERGE cohort and focused on the first 2 PCs (hereafter referred to as phenotype PCs), which explained in total 63.8% variances. Among these, PC1 explained 45.02% variances, followed by PC2 at 18.82%. We fit linear models using age, sex, sites, and the first 10 genetic PCs calculated for these 100,272 patients to evaluate the contribution to each phenotype PC. Then, we performed ANOVA to evaluate the variance explained by each covariate (Table 4). In general, all covariates contribute to the variances of phenotypes. Age contributes the most in PC1 and sex in PC2. The linear model and ANOVA are implemented using the Python package statsmodels^104^, version 0.12.2. For a complete view, we also presented results of PC3-5 (explained 22.15% variance in total) in the Supplementary Table 5.

### Random sampling to predict phenotype distances

Besides PCA analysis evaluating the contribution of age, sex, and genetics to phenotype development, we performed this bootstrapping sampling strategy to investigate how these components can explain the variances seen in phenotype differences of paired patients. To start, we performed three rounds of random sampling without replacement, with each round consisting of 100, 500, and 1000 patients from the eMERGE cohort. We then paired two patients in each round, yielding 4945 (${C}_{100}^{2}$), 124,750 (${C}_{500}^{2}$), and 499,500 (${C}_{1000}^{2}$) pairs. For each pair, we quantified their phenotype distances and employed a linear model to evaluate the contributions of age gaps, sex differences, sites, and genetic distances to their phenotype distances. The phenotype distance between a pair of patients is computed as the Euclidean distance of the embedding values (derived from our previous work^10^), which represent the digital phenotype of each individual. The sex difference is a binary variable with 1 indicating a different sex and 0 indicating the same sex. The genetic distance is the Euclidean distance of the first 10 genetic PCs. In detail, for each pair of patients_*i*_ and patients_*j*_, we have:

$$\mathrm{phenotype}\,{\mathrm{distance}}_{i,j}=\mathrm{age}\,{\mathrm{gap}}_{i,j}+\mathrm{sex}\,{\mathrm{diff}}_{i,j}+\mathrm{site}\,{\mathrm{diff}}_{i,j}+\mathrm{genetic}\,{\mathrm{distance}}_{i,j}$$

gWhere age gap is $\mathrm{age}\,{\mathrm{gap}}_{i,j}=\left|{\mathrm{age}}_{i}-{\mathrm{age}}_{j}\right|$. Sex diff as binary variable, where

$${\left({sex}\,{diff}\right)}_{i,j}=\left\{\begin{array}{c}0,\,\left({se}{x}_{i}={\rm{s}}e{x}_{j}\right)\\ 1,\,\left({se}{x}_{i}\ne {se}{x}_{j}\right)\end{array}\right.$$

Similar for sites,

$${\left({site}\,{diff}\right)}_{i,j}=\left\{\begin{array}{c}0,\,\left({{site}}_{i}=\,{{site}}_{j}\right)\\ 1,\,\left({{site}}_{i}\ne {{site}}_{j}\right)\end{array}\right.$$

The phenotype distance and genetic distance are both Euclidean distance: $d\left({\mathrm{embedding}}_{i},{\mathrm{embedding}}_{j}\right)=\left|\left|{\mathrm{embedding}}_{i}-{\mathrm{embedding}}_{j}\right|\right|$, and $d\left({\mathrm{geneticPC}}_{i},{\mathrm{geneticPC}}_{j}\right)=\left|\left|{\mathrm{geneticPC}}_{i},-{\mathrm{geneticPC}}_{j}\right|\right|$

The linear model is implemented using Python package statsmodels^104^, version 0.12.2.

### Clustering of UW cohort and the identification of high-risk clusters

We applied the GMM trained on the eMERGE dataset directly to the randomly selected 100 K UW cohort without fine-tuning. We examined the high-risk cluster group (clusters 67, 38, 5, 66, 34, 28, 52, 1, 64) identified in the eMERGE dataset and expected them to have a higher mortality rate in the UW cohort (reproducibility). As this is a retrospective observation, we compared the mortality rate differences between the high-risk clusters and others. In total, 4.9% of the randomly selected 100K UW patients are deceased and used as a baseline mortality rate. The high-risk clusters (clusters 67, 38, 5, 66, 34, 28, 52, 1, 64) have a mortality rate of 16.35%, with an odds of 4.21 (*p* = 4.02 × 10^−152^) compared to non-high-risk clusters of a 4.44% mortality rate. Knowing this could be potentially biased by age, as we showed age and sex are driving the clustering membership, we adjusted for sex and age in a linear model predicting the mortality, which still yielded a significantly higher risk for those high-risk clusters (odds = 2.65, *p* = 1.32 × 10^−82^).

### Cross-phenotype association analysis within clusters

To identify cross-phenotype association within clusters, we downloaded the GWAS summary statistics from the GWAS catalog (https://www.ebi.ac.uk/gwas/) and the trait mapping file (https://ftp.ebi.ac.uk/pub/databases/gwas/releases/latest/gwas-efo-trait-mappings.tsv). We used the mapping file to convert the annotated phenotype to phecodes. For each cluster, we evaluated if two significantly enriched phenotypes (*p*-values < 0.05 after Bonferroni correction) in this cluster have at least one shared genetic association documented from the GWAS catalog; we then defined it as an observed cross-phenotype association. We plotted the observed cross-phenotype association in the network format, with nodes indicating each phenotype and an edge connecting them representing observed cross-phenotype association (Fig. 4e). To statistically evaluate if enriched phenotypes are more likely to have shared genetic associations, we performed Fisher’s exact test for each cluster to investigate the odds of observing such an event. Specifically, we divide phenotypes into four categories: (a) enriched and has shared genetic associations with other phenotypes, (b) not enriched while sharing a genetic association with other phenotypes, (c) enriched and not sharing any genetic associations with other enriched phenotypes, (d) not enriched and not sharing any genetic associations with other phenotypes. Then four of these events (a–d) are used to form the 2 × 2 table and are used to compute the odds. Consistently, all clusters showed a higher odds of observing enriched phenotypes having shared genetic associations, with 57 out of 70 statistically significant results after adjusting for Bonferroni correction.

### Heritability (h2) analysis and phenotype mapping

We downloaded the heritability (h2) dataset from Jia et al.^54^. We mapped the 285 unique phenotypes with h2 information to phecodes (Supplementary Table 6). We then calculated the median onset time of these 285 phenotypes and performed a Spearman correlation analysis (scipy^102^ version 1.7.1). We find strong negative correlations between phenotype onset median onset time and h2 (Spearman *ρ* = −0.37, *p* = 4.87 × 10^−12^).

### Modeling of pseudo-aging rate using longitudinal patient embeddings

To investigate the rate of aging, we adapted the concept from Tian et al.^72^ by defining the *age gap* as the difference between predicted age and true chronological age in longitudinal data. Predicted age reflects the model’s estimate of a patient’s age based on embedding features, and thus, the age gap serves as a proxy for aging rate. We analyzed all longitudinal events of 17,395 eMERGE participants with more than 20 years of longitudinal data, using 80% for training and 20% for testing. A gradient boosting regressor was trained on embedding features to predict chronological age, with performance evaluated on the held-out test set. To ensure balanced representation and prevent overfitting to specific age ranges, we randomly sampled 20,000 patients per birth decade within the training set (Fig. S10a). For each patient, we averaged the age gap across all longitudinal predictions to derive a pseudo-aging rate at the individual patient level.

### Regression analysis of aging rate predicting lab results

We performed linear regression for individual labs, including lymphocyte (OMOP id 3004327), platelet count (OMOP id 3007461, 3024929), LDL (OMOP id 3035009, 3028288, 3035899, 3009966, 3028437, 3004169, 3053341, 40758569, 3033200, 3001308, 3038988, 42870529), HDL (OMOP id 3007070), fasting glucose (OMOP id 3037110), and triglycerides (OMOP id 3022192). For each model, we used the aging rate (mean age gap) of individuals to predict the lab, including sex, age, sites, and genetic ancestry race as covariates. For each lab in lymphocyte, platelet count, LDL, HDL, fasting glucose, and triglycerides, we performed the regression analysis using statsmodels^104^ with the following formula (for individual patient_*i*_):

$$\mathrm{lab}=\mathrm{pseudo}-\mathrm{aging}\,{\mathrm{rate}}_{i}+{\mathrm{age}}_{i}+{\mathrm{sex}}_{i}+{\mathrm{site}}_{i}+\mathrm{genetic}\,\mathrm{ancestry}\,{\mathrm{race}}_{i}$$

### Calculating longevity polygenic risk score (PRS)

The longevity PRS is calculated using plink software. We first downloaded the longevity PRS (PGS000906) from the PGS catalog^108^. We downloaded the beta coefficient and variants then used the plink –score function^109^ to calculate the PRS score for all 100,272 participants in the eMERGE cohort.

### Multi-category phenotype enrichment in successfully aged population

We mapped phecodes to the phenotype categories (e.g., essential hypertension is mapped to the circulatory system) using the PheWAS R package^110^ and compared the numbers of unique phenotype categories between successfully aged participants versus general participants during their 30s, 40s, 50s, and 60s (Fig. 6d) using Wilcoxon rank-sum test. We avoid comparing the age of the 70s because of potential biases, as the successfully aged population is defined as those with an age greater than or equal to 75.

### Phenotype onset time comparison between successfully aged and general population

We compared phenotype onset times between successfully aged participants and the remaining population using linear regression models, adjusting for age, sex, study site, and genetic ancestry. For each of the 82 mortality-associated phenotypes, we defined a binary variable population indicating whether a patient belonged to the successfully aged group (population_*i*_ = 1) or the general population (population_*i*_ = 0). The regression analyzes were performed with the statsmodels package in Python using the following formula^104^:

$$\mathrm{phenotype}\,\mathrm{on}\,\mathrm{set}\,{\mathrm{age}}_{i}={\mathrm{population}}_{i}+{\mathrm{age}}_{i}+{\mathrm{sex}}_{i}+{\mathrm{site}}_{i}+\mathrm{genetic}\,\mathrm{ancestry}\,{\mathrm{race}}_{i}$$

### Simulation of population structure of aging

We performed a simple simulation experiment to test our hypothesis—the observed discrepancy in pseudo-aging rate and genetic longevity score indicates survival selection, and resulted in heterogeneous population structures. To demonstrate this, we first simulated a uniform sample (*N*_*total*_ = 100,000) of samples with true biological age between 30 and 90. We then defined another variable, observed age (chronological age), that is a linear function of biological age with Gaussian noise, represented as $\text{observed age}=\text{biological age}+{\rm{\epsilon }}$, where ${\rm{\epsilon }}\sim N\left(\mathrm{0,10}\right)$. Then, for each decade, we simulate the selection process by randomly sampling *n* participants and subtracting their biological age by a predefined offset ${\rm{\lambda }}=\,3.5$ and the current decade *i*, which is $\text{biological age}-{\rm{\lambda }}-i$. Through this process, the selected *n* participants are going to have a younger biological age than their observed age. The number of selected participants *n* is a function of age (a function of selection, the older the age, the stronger the selection effect), where $n=i\cdot \frac{{e}^{i+1}}{c}$, where *i* represents the current decade of patient age (e.g., 3, 4, 5) and *c* is a constant offset to normalize the growth rate of *n*, which is set as *c* = 350. We choose *n* to be an exponential function of the decade *i* as the selection shall be similar to the selection of cellular growth. Then in the modeling parts, we do not allow a direct measurement of biological age (the unknown truth), so we defined an observed variable as a biological marker that is a linear function of biological age with Gaussian noise $\text{biological marker}=\text{biological age}+\,{\rm{\epsilon }}$, where $\epsilon \sim N\left(\mathrm{0,10}\right)$. This variable represents the biomarkers or features we used to model age. In this setup, we then implemented the same gradient boosting regressor (Scikit-learn version 0.24.2) to model the observed age, using the formula:

$$\text{observed age}={\rm{\beta }}\,\cdot \mathrm{biological}\,\mathrm{market}$$

The results are shown in Fig. S10e, f, where we show that younger participants are commonly predicted to be older while older participants are predicted younger.

## Supplementary information

Supplementary Information

## Data Availability

The datasets generated and/or analyzed during the current study are not publicly available due to the protection of patient privacy and compliance with regulations, but are available from the eMERGE network (https://emerge-network.org/) on reasonable request.

## Code availability

Code to produce the results is posted on the GitHub page: https://github.com/suxian06/patient-topic-modeling.
