# Supplementary material for: Unsupervised characterization of 100,272 EHR patients identifies high-risk groups and comorbidities linked to premature aging
Source: NPJ Digit Med. 2026 Jun 23;9:682. doi: 10.1038/s41746-026-02913-x (PMC13554212; doi:10.1038/s41746-026-02913-x)
Supplement: Supplementary file 1 [file 41746_2026_2913_MOESM1_ESM.pdf]

Supplementary information

Supplementary Figure 1. Evaluation metrics for K-means and GMM clustering methods.

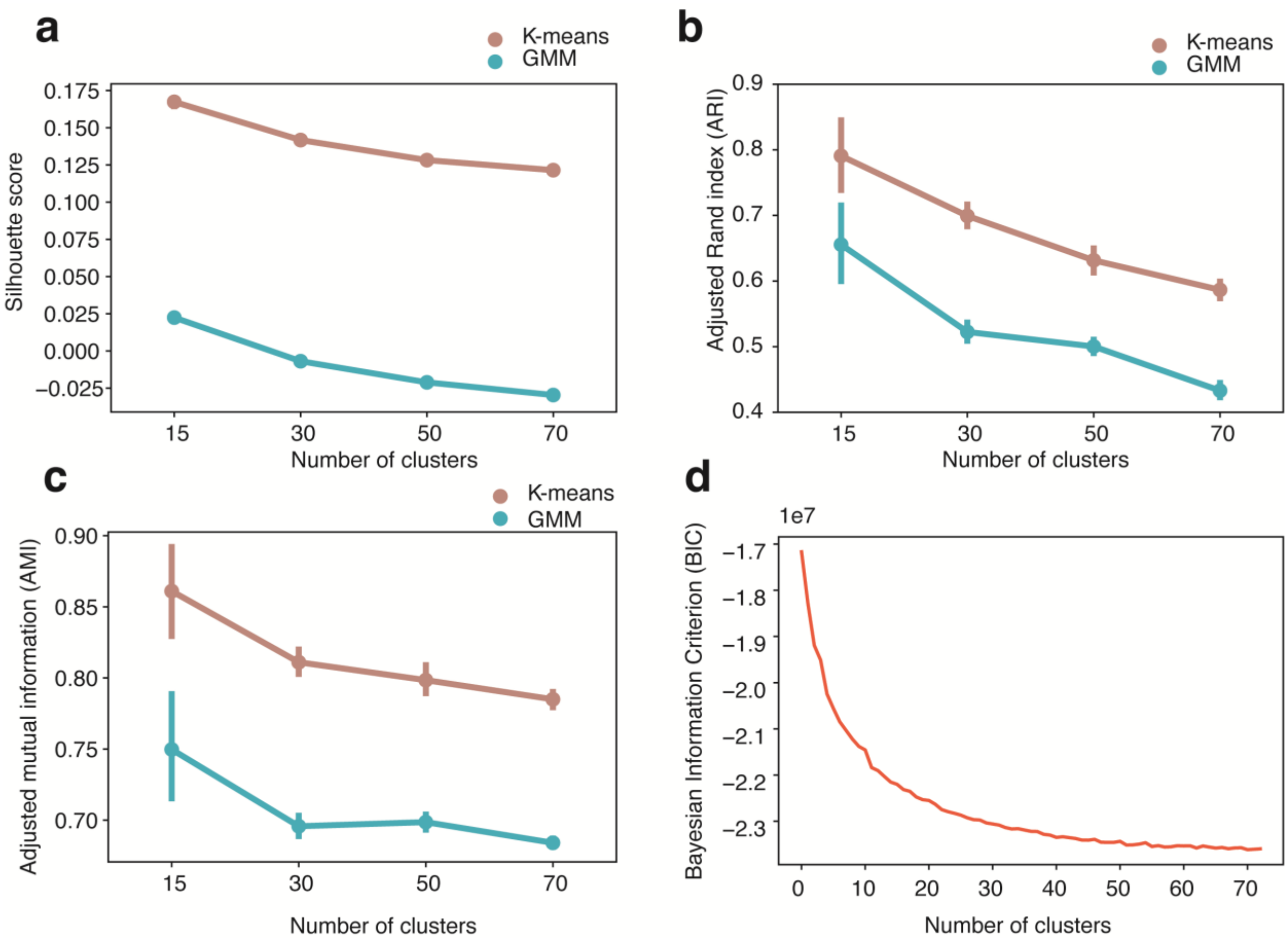

a. Silhouette score evaluation on K-means (brown) and GMM (blue) for different cluster numbers (the x-axis).

b. Adjusted rand index (ARI) score evaluation on K-means (brown) and GMM (blue) for different cluster numbers (the x-axis).

c. Adjusted mutual information (AMI) score evaluation on K-means (brown) and GMM (blue) for different cluster numbers (the x-axis).

d. BIC reported on different clusters of GMM.

Supplementary Figure 2. Clusters form topological-free network structures

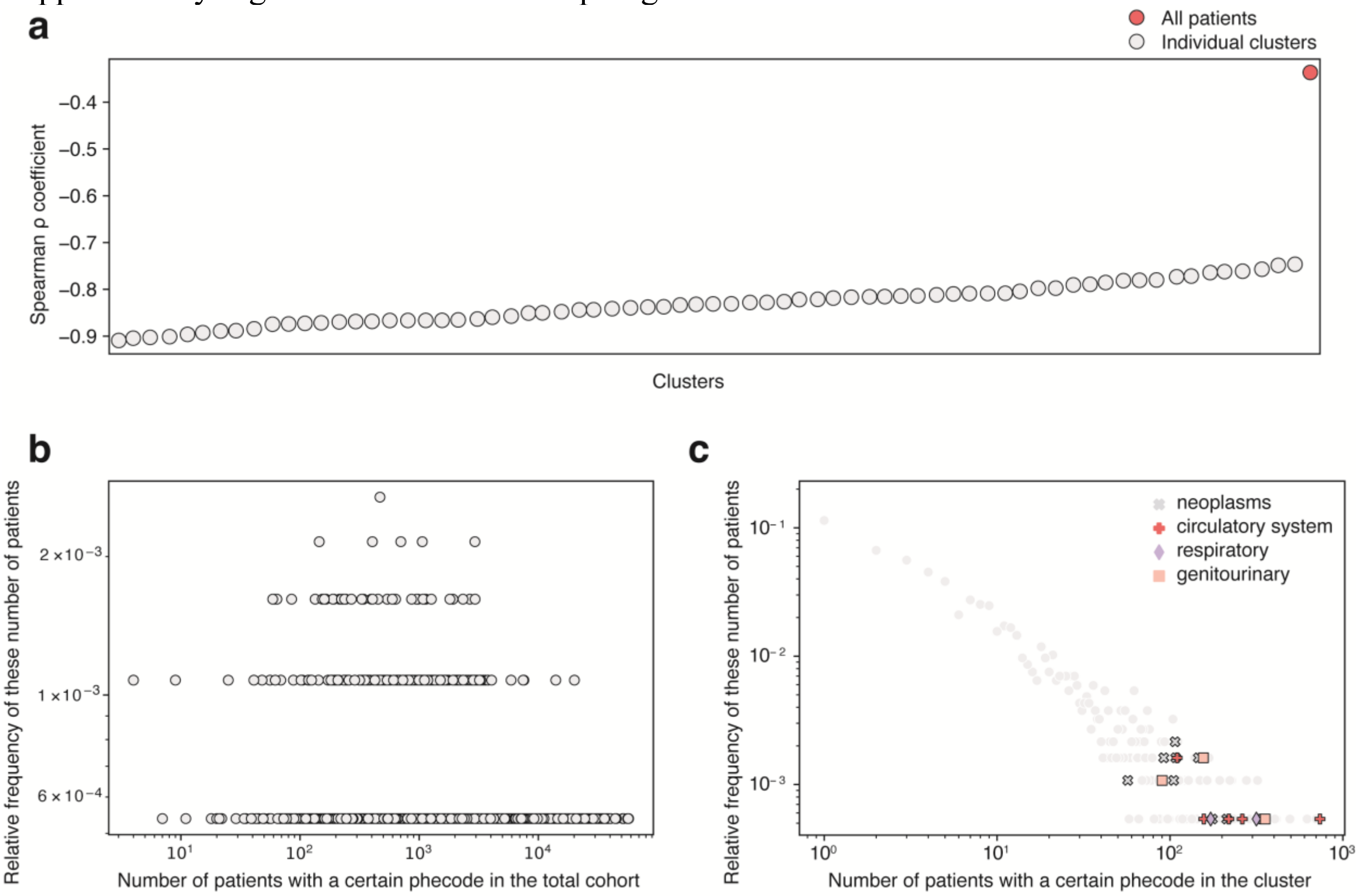

a. Spearman correlation coefficient between the number of patients having the phenotypes within a specific cluster and the frequency of the corresponding number of patients in 70 clusters (gray dots) and the whole cohort (red dot).
b. Scatter plot showing the relationship between the number of patients having the phenotypes within the whole cohort (the x-axis) and the frequency of the corresponding number of patients (the y-axis).
c. Scatter plot showing the relationship between the number of patients having the phenotypes within the cluster (the x-axis) and the frequency of the corresponding number of patients (the y-axis), illustrated on cluster 47. Colored marks indicate the category of the top 20 enriched phenotypes in this cluster, with each color code denoting the phenotype category.

Supplementary Figure 3. The onset time of phenotypes can accurately predict patient age.

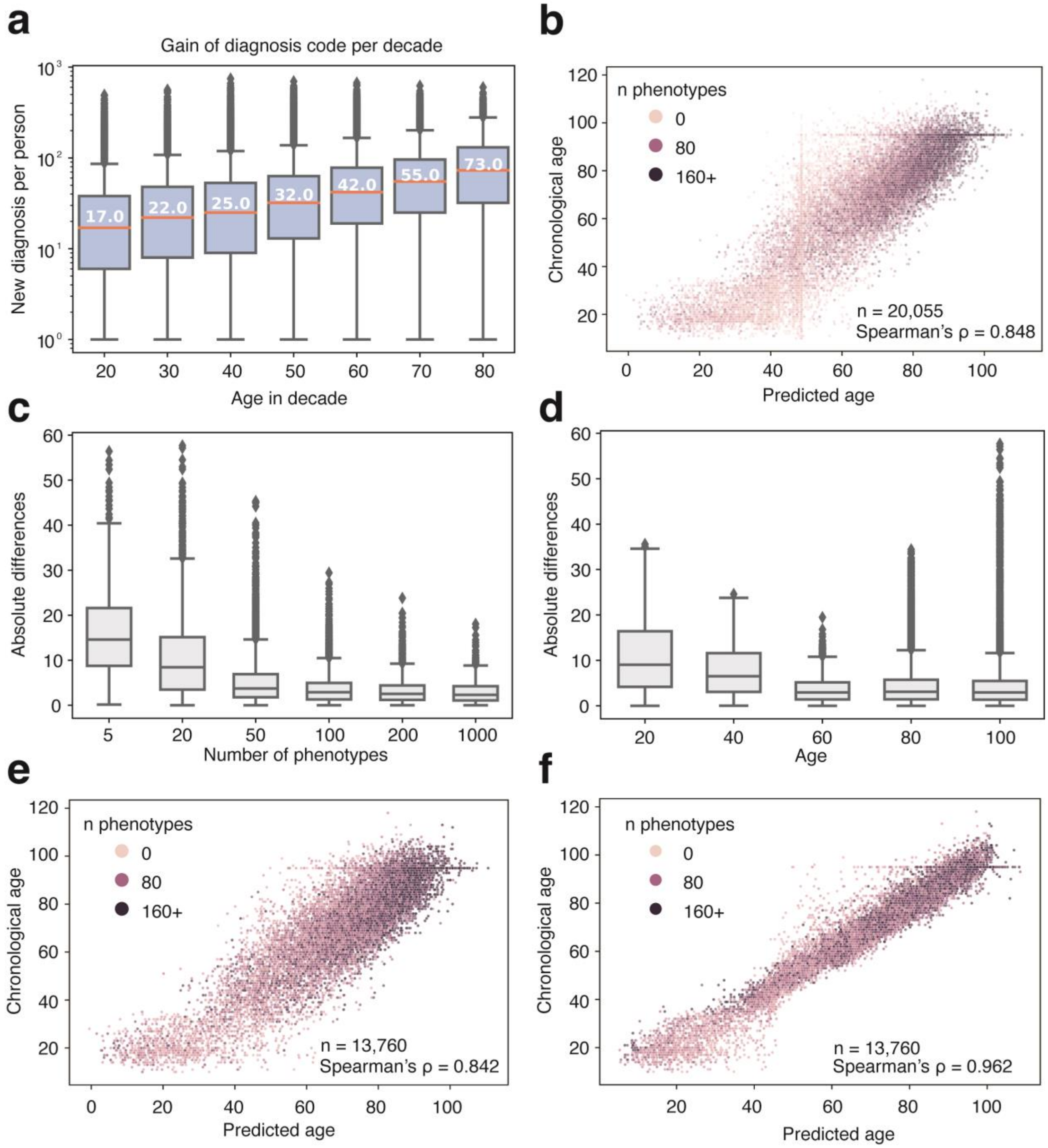

a. Boxplots showing the relationship between chronological age (the x-axis) and the distribution of new diagnoses each decade (the y-axis) per person.

b. Scatterplot showing the relationship between predicted age (the x-axis) and the chronological age (the y-axis) for each patient in the test set (n = 20,055), using binary features (presence vs absence of phenotypes) colored by the number of unique phenotypes they have.

c-d. Boxplots showing the distribution of absolute differences in the model prediction, stratified by the number of phenotypes an individual patient has (c) and the chronological age of the patient (d).

e-f. Scatterplot showing the relationship between predicted age (the x-axis) and the chronological age (the y-axis) for each patient in the test set that has at least 30 numbers of unique phenotypes (n = 13,760), using binary features (e), and using phenotype onset time (f), colored by the number of unique phenotypes they have.

Supplementary Figure 4. Shapley value of top features in modeling of patient age predictions.

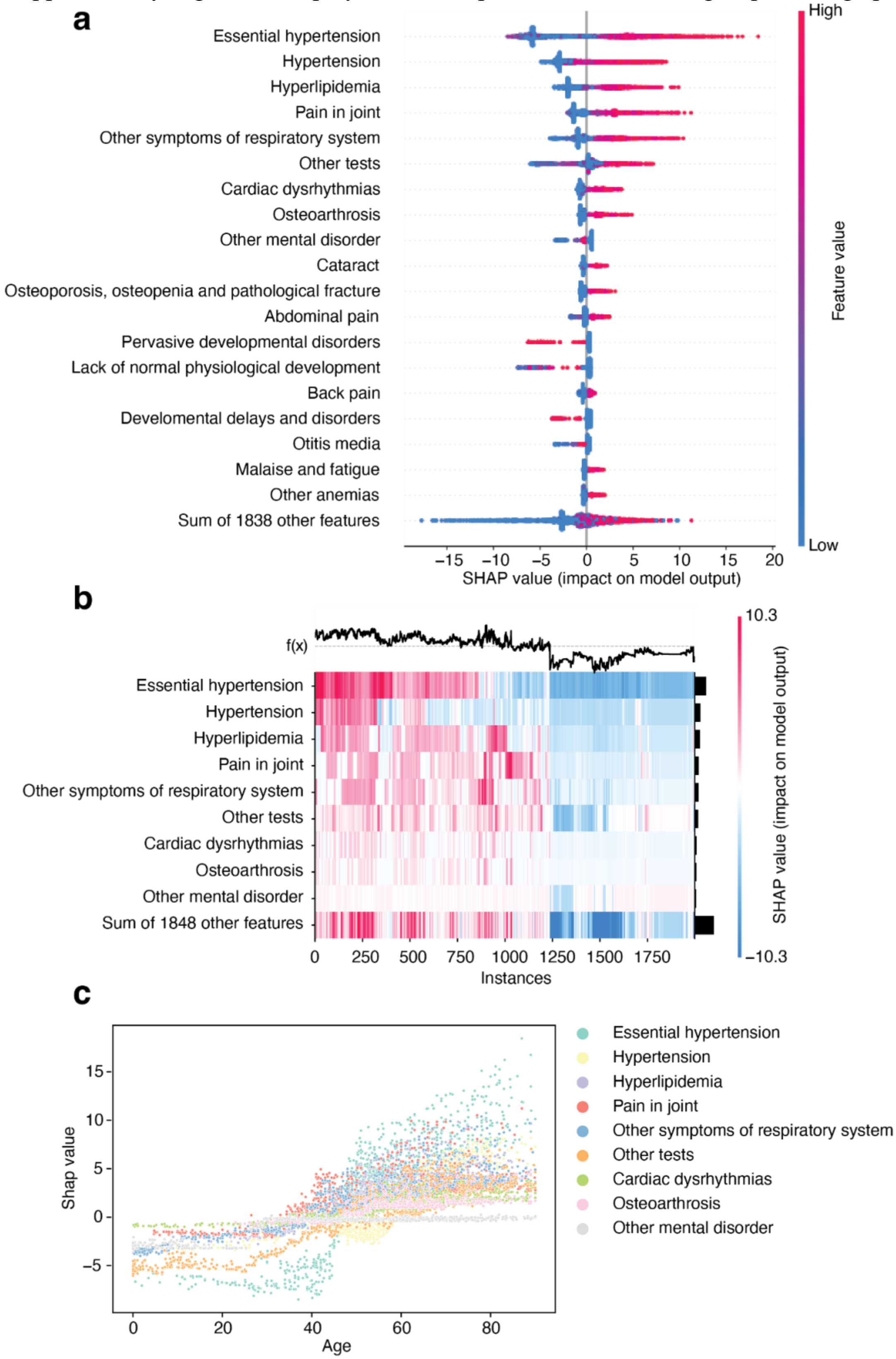

a. Stripplot showing the contribution of top features (the x-axis) in the GBT model predicting age, colored by the feature value.
b. Clustered heatmap showing the top features, colored by their SHAP value, with the model output f(x) on the top.
c. Scatterplot showing the SHAP value (the y-axis) of these top features across different ages (the x-axis).

Supplementary Figure 5. Sex contributes to phenotype frequency and onset discrepancies.

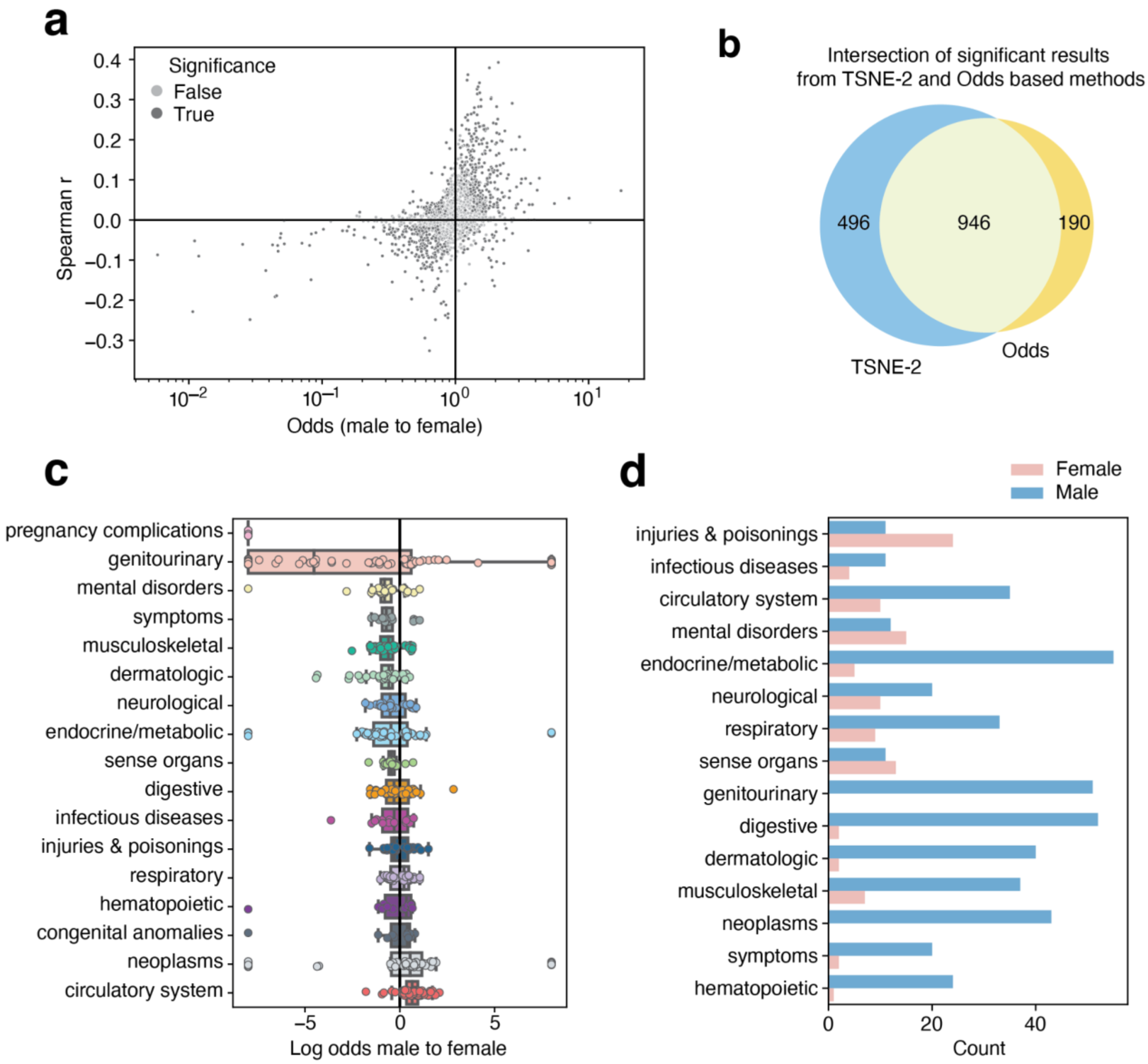

a. Scatterplot showing the concordance of two different methods identifying discrepancies in phenotype prevalence, with the x-axis denoting the odds ratio of observing each phenotype in male versus in female and the y-axis indicating the Spearman correlation coefficient between TSNE-2 and each phenotype.

b. A venn diagram indicating the overlap between the two different methods.

c. Scatterplot showing the prevalence differences of phenotypes between males and females, with computed odds in the x-axis and grouped by the phenotype categories in the y-axis. All log odds with absolute value greater than 10 were capped at 10 for visualization convenience.

d. Barplot showing the initial results of phenotype onset time differences between males and females, without adjusting for the number of hospital visits and the age at first hospital visit. The length of the bar indicates the number of phenotypes that have a later onset than the opposite biological sex, with blue indicating late onset in males and pink representing late onset in females.

Supplementary Figure 6. Principal components of the embeddings and their association with age, sex, and genetics.

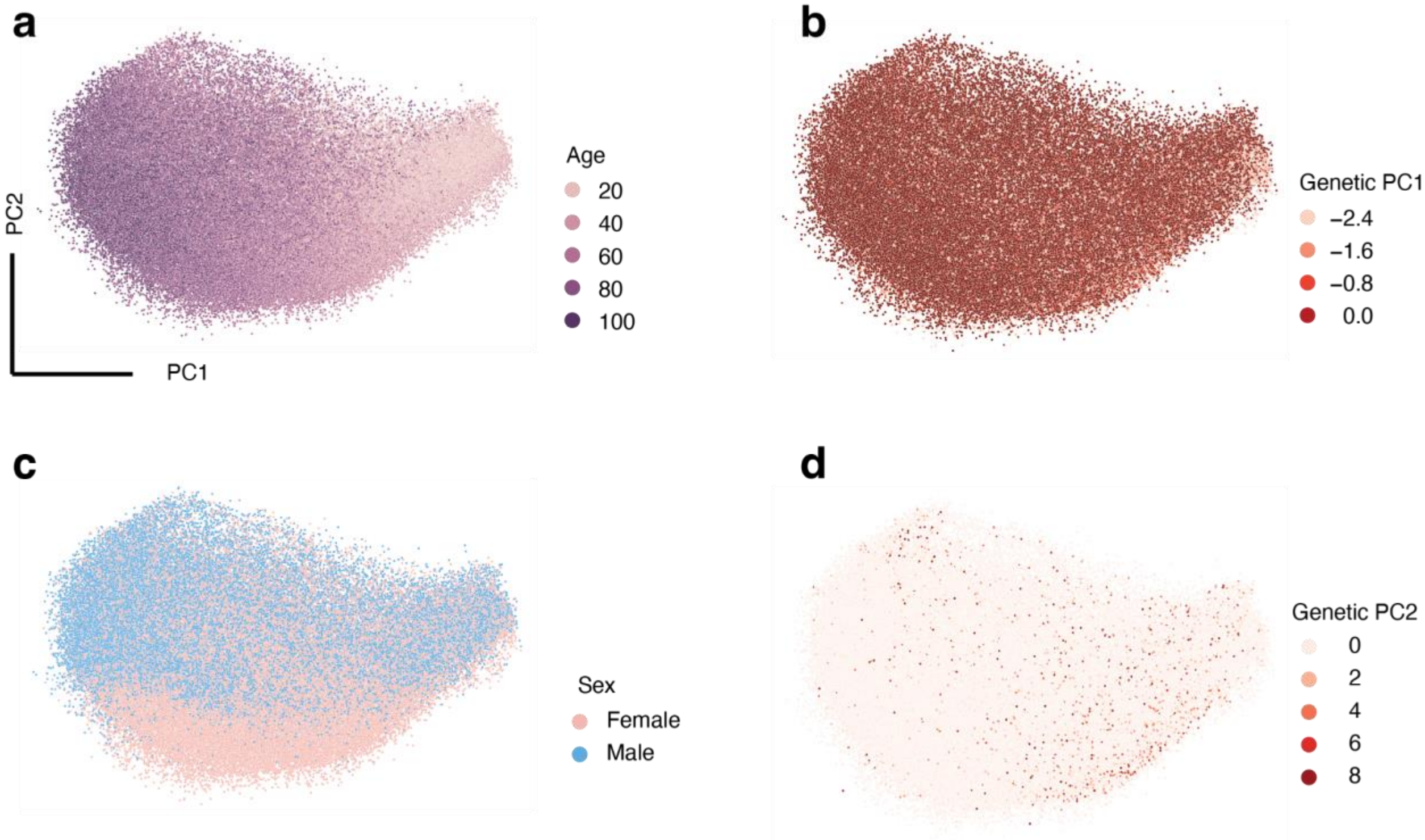

a-d. Scatterplots showing PC1 and PC2 of the digital phenotypes (embeddings) colored by patient age (a), genetic PC1 (b), patient biological sex (c), and genetic PC2 (d).

Supplementary Figure 7. Phenotype enrichment for high CCI clusters.

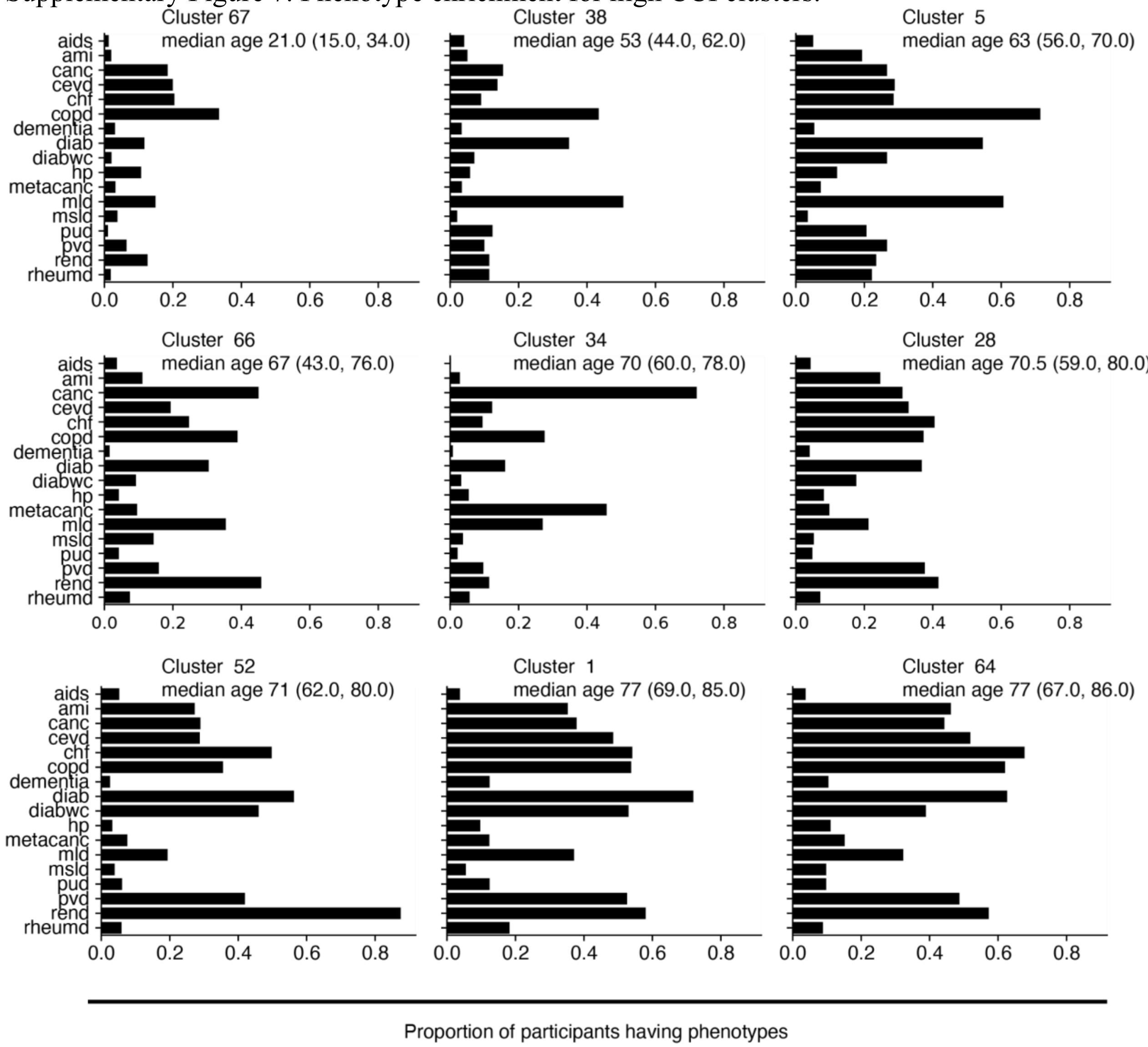

Supplementary Figure 8. Validating the high-risk cluster group in UW cohort.

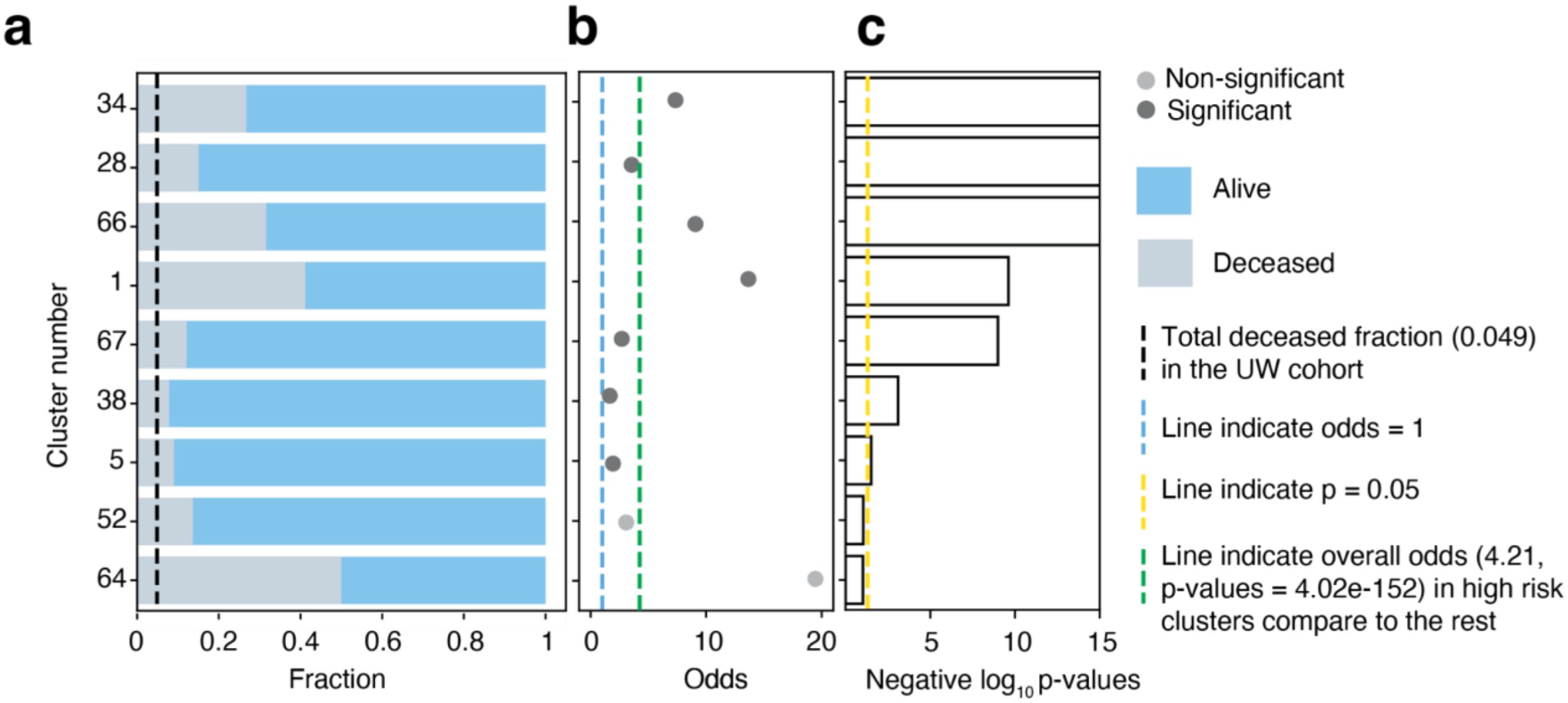

a. Barplot indicates the fraction of alive (blue) and deceased (gray) patients in the high-risk clusters (the y-axis) of the UW validation cohort, with a dark line denoting the baseline rate of mortality in the UW validation cohort.

b. Stripplot denoting the hazard ratio between each cluster (the y-axis) and the rest of the UW cohort, with darker dots indicating significant results and lighter dots non-significant results. The green line dictates the overall hazard ratio of all high-risk clusters compared to the rest of the UW cohort.

c. Barplot showing the negative $\log_{10}$ p-values of the hazard ratio in (b), with the yellow line indicating p-values of 0.05.

Supplementary Figure 9. Top 20 phenotypes that showed significant association between their onset time and CCI in eMERGE cohort (a); and mortality in the UW cohort (b).

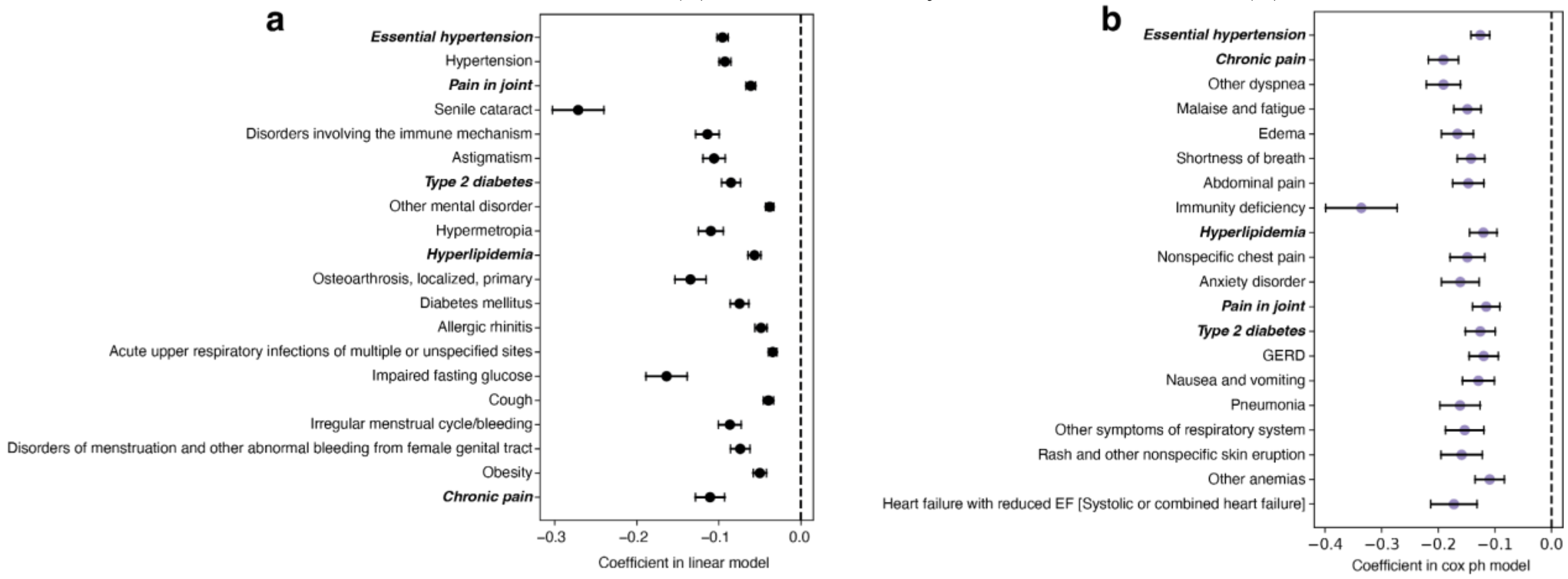

a-b. Stripplot showing the top 20 phenotypes with their onset time significantly associated with CCI in the eMERGE cohort (a) and the top 20 phenotypes with their onset time significantly associated with mortality in the UW cohort (b).

Supplementary Figure 10. Illustration and modeling of aging speed.

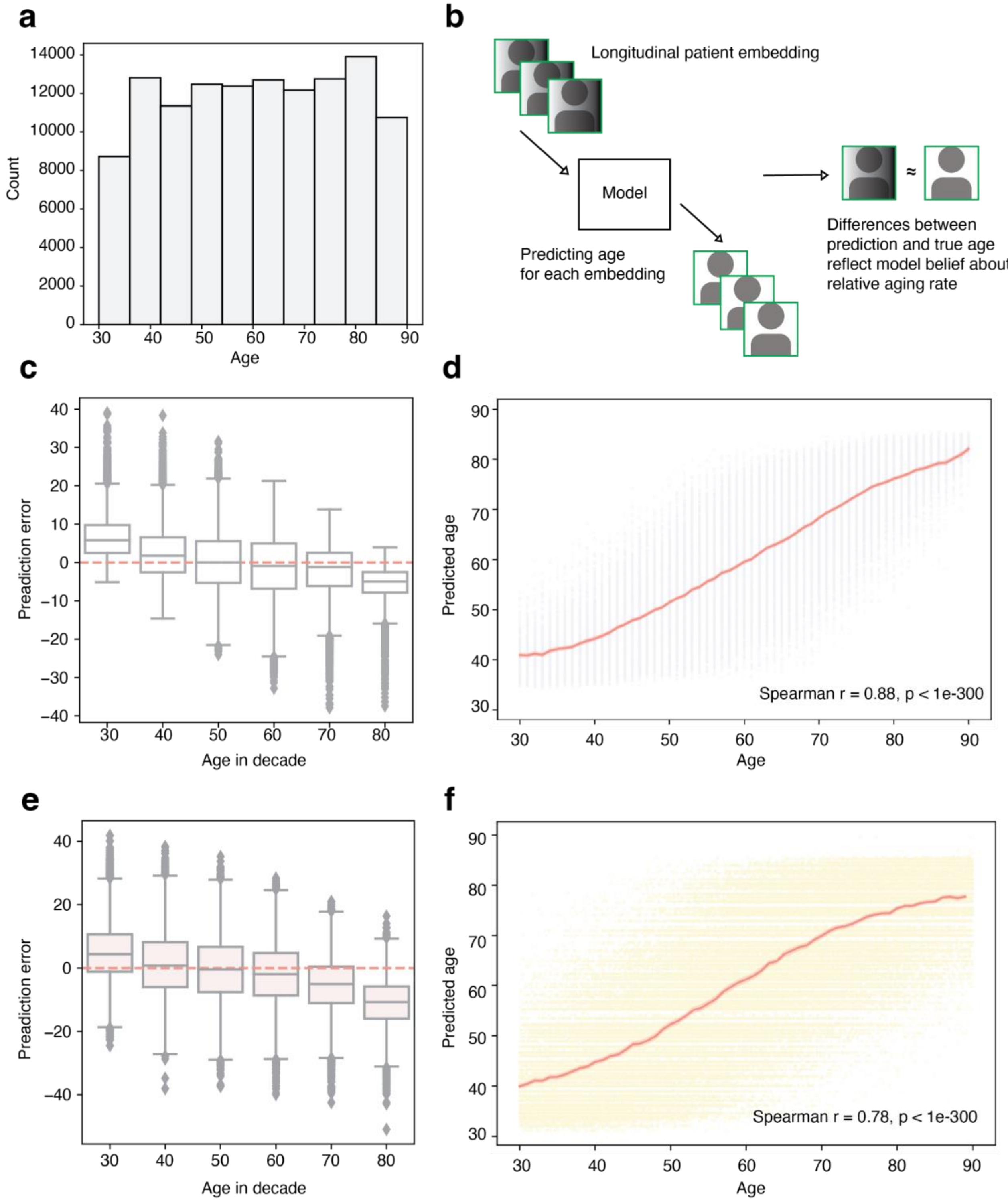

a. Histogram showing the distribution of selected training samples in the model used to predict age using embedding features after sampling using a uniform distribution strategy.
b. Illustration of the definition of pseudo-aging rate, defined as the differences between predicted age and current age of a patient.

c. Barplot showing the distribution of prediction error in the y-axis (the differences between predicted age and current age of a patient) across different age groups in the x-axis.
d. Scatterplot showing the relationship between the predicted age and the true age, with a red line showing the mean value of the prediction.
e-f. The same plot as c and d but using simulation experiments (see Methods).

Supplementary Figure 11. Reproducing pseudo-aging results in Figure 6 after adjusting for the number of years of follow-up.

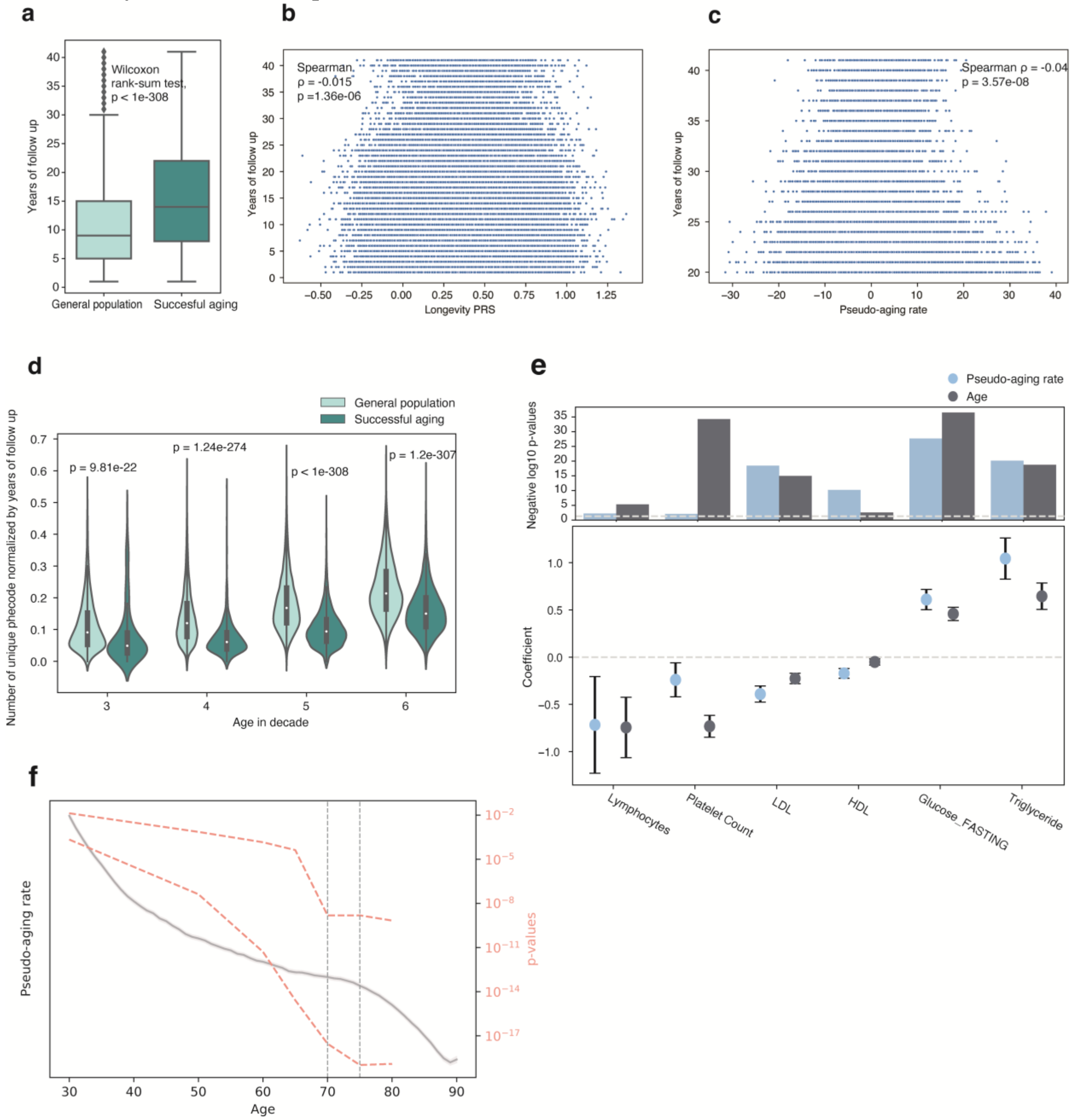

a. Boxplot showing the number of years of follow-up differences between successfully aged populations and the general population in the eMERGE cohort.
b-c. Scatterplot showing correlations between the number of years of follow-up versus longevity PRS (b) and number of years of follow-up versus pseudo-aging rate (c).
d. Violin plot showing the number of phenotype categories (the y-axis) individuals have at a specific age group in decades (the x-axis), normalized by the number of years of follow-up, as an adjusted version of Figure 6d.
e. Barplots and stripplots showing model coefficients and p-values of aging rate predict labs that are known to be associated with aging. The bottom is a point plot with a confidence interval

showing the coefficient of age rate and age in linear models adjusted for sex, sites, and genetic ancestry, predicting each lab respectively in the corresponding x-axis. The top bar plot represents the negative log10 p-values of each lab. This is the same analysis of Figure 6b adjusted for the number of years of follow-up.
f. Replotting Figure 6c adjusted for the number of years of follow-up. A line plot illustrating how the pseudo-aging rate and genetic longevity jointly revealed survivorship bias in patients aged over 75. The grey line represents the average aging rate across 17,395 patients in the eMERGE cohort with longitudinal data. For each point along the x-axis, the two pink dotted lines display p-values comparing genetic longevity PRS for individuals older than the given age versus those of the same age or younger. The upper pink dotted line corresponds to the subset with longitudinal data, while the lower line reflects the full cohort of 100,272 eMERGE patients. For each point along the x-axis, p-values compare individuals older than the given age with those of the same age or younger.

Supplementary Figure 12. Comparing K-means clustering with GMM.

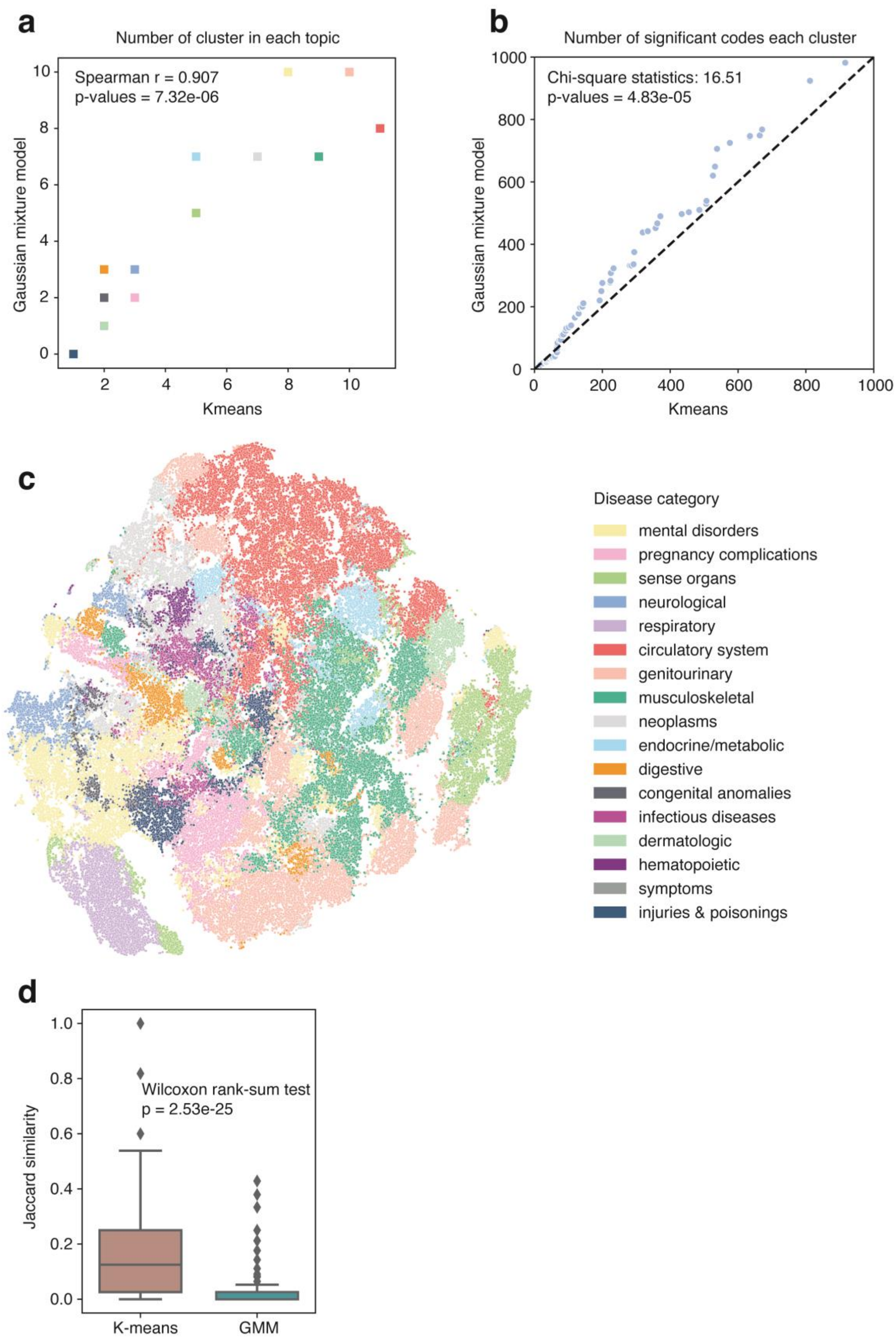
a
Number of cluster in each topic
Spearman r = 0.907
p-values = 7.32e-06
Gaussian mixture model
Kmeans
b
Number of significant codes each cluster
Chi-square statistics: 16.51
p-values = 4.83e-05
Gaussian mixture model
Kmeans
c
Disease category
mental disorders
pregnancy complications
sense organs
neurological
respiratory
circulatory system
genitourinary
musculoskeletal
neoplasms
endocrine/metabolic
digestive
congenital anomalies
infectious diseases
dermatologic
hematopoietic
symptoms
injuries & poisonings
d
Wilcoxon rank-sum test
p = 2.53e-25
Jaccard similarity
K-means
GMM

a. Pointplot showing the number of clusters identified in each phenotype category using the Gaussian mixture model (the y-axis) and K-means clustering (the x-axis), with each colored dot representing a phenotype category.
b. Scatterplot showing the number of significantly enriched phenotypes in each cluster identified using the Gaussian mixture model (the y-axis) and K-means clustering (the x-axis). The chi-square test represents the test statistic of the hypothesis that the two models should identify a similar number of enriched codes in these clusters.
c. Scatterplot showing TSNE visualization of patients colored by phenotype categories based on the K-means clustering model.
d. Boxplot showing the Jaccard similarity of pair-wise comparison between clusters' overlapped phenotypes in K-means (brown) and GMM (blue).

Supplementary Figure 13. Density plots showing the distributions of embedding features.

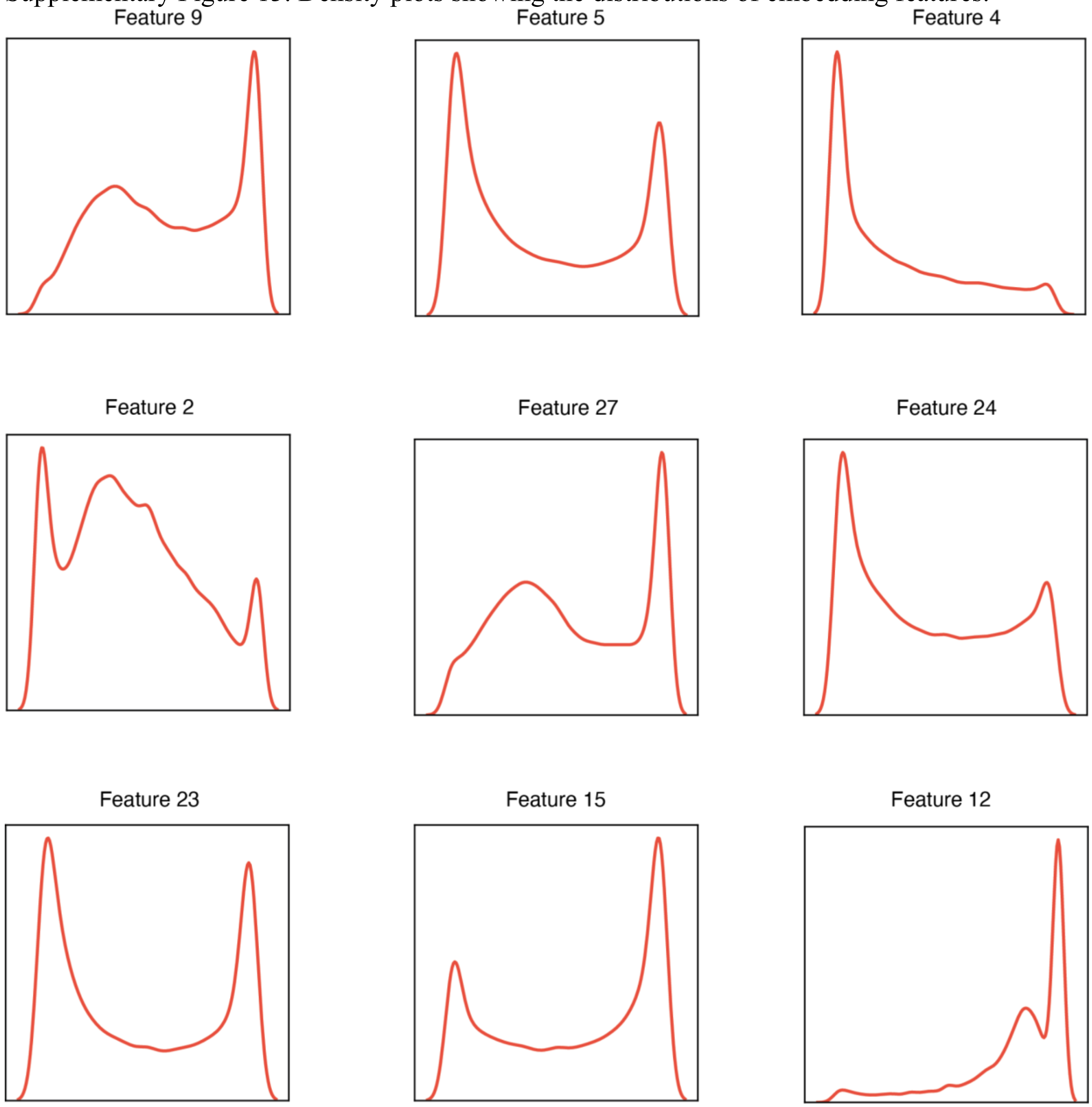

Supplementary Table 1. Fisher's exact test of the relationship between enriched phenotype in pleiotropic phenotypes in individual clusters.

Odds	p-values	FDR_BH	Bonferroni ($p < 0.05$)	Cluster
4.76914085	1.19E-07	1.78E-07	TRUE	37
5.37853107	0.03586215	0.03803562	FALSE	69
6.75925926	1.11E-06	1.55E-06	TRUE	19
4.12820513	4.81E-06	6.61E-06	TRUE	65
3.83974824	6.03E-19	2.22E-18	TRUE	59
4	4.19E-09	7.16E-09	TRUE	35
4.19818064	8.45E-12	1.74E-11	TRUE	18
4.65477294	1.55E-22	1.81E-21	TRUE	61
	1	1	FALSE	22
4.56394539	4.93E-10	9.08E-10	TRUE	30
4.35576739	8.30E-22	7.26E-21	TRUE	15
3.90470172	6.31E-09	1.05E-08	TRUE	12
3.62546153	3.42E-10	6.47E-10	TRUE	17
7.07692308	9.65E-08	1.50E-07	TRUE	0
5.02713704	4.89E-25	1.71E-23	TRUE	31

4.70967742	6.10E-20	2.67E-19	TRUE	25
4.30024213	0.00773409	0.00832902	FALSE	4
4.36363636	0.00106822	0.00126737	FALSE	11
4.8125	1.07E-07	1.62E-07	TRUE	24
5.19047619	0.00575115	0.00629032	FALSE	58
5.27901786	0.00074956	0.00090464	FALSE	3
3.98409191	4.11E-12	8.71E-12	TRUE	67
3.6984739	3.88E-18	1.29E-17	TRUE	27
5.76808835	2.15E-17	6.54E-17	TRUE	7
4.13785047	8.57E-16	2.31E-15	TRUE	6
5.22263682	4.07E-05	5.18E-05	TRUE	29
4.00589102	1.05E-20	5.26E-20	TRUE	46
5.33391437	4.04E-23	5.66E-22	TRUE	2
5.33769063	4.20E-11	8.16E-11	TRUE	48
3.44303797	0.22473515	0.23479791	FALSE	20
4.09710675	1.33E-07	1.90E-07	TRUE	47
3.86837558	1.26E-16	3.68E-16	TRUE	42

8.48484848	0.0002394	0.00029925	TRUE	44
4.43202208	8.35E-15	2.16E-14	TRUE	28
4.36528029	1.60E-11	3.19E-11	TRUE	62
6.11084337	0.00204417	0.00238487	FALSE	55
4.42735043	1.31E-07	1.90E-07	TRUE	60
4.72910217	1.69E-24	3.95E-23	TRUE	63
5.25122021	4.73E-24	8.28E-23	TRUE	45
5.55775076	1.63E-19	6.35E-19	TRUE	13
5.27025898	1.32E-25	9.21E-24	TRUE	1
4.54191033	5.29E-08	8.42E-08	TRUE	14
	1	1	FALSE	21
4.8403863	3.09E-22	3.09E-21	TRUE	38
7.64367816	0.00279438	0.00315494	FALSE	68
3.47757671	4.49E-16	1.26E-15	TRUE	41
5.39087657	5.39E-06	7.25E-06	TRUE	10
3.96919192	3.67E-09	6.58E-09	TRUE	33
3.14473684	0.2517879	0.25919343	FALSE	8

5.13606676	6.51E-21	3.80E-20	TRUE	39
4.82181448	6.97E-13	1.57E-12	TRUE	57
4.00029125	3.21E-18	1.12E-17	TRUE	16
4.0611729	6.18E-21	3.80E-20	TRUE	5
3.70784314	3.87E-09	6.78E-09	TRUE	34
4.4682861	5.42E-13	1.27E-12	TRUE	49
4.45546737	1.45E-21	1.13E-20	TRUE	43
5.08260325	7.16E-20	2.95E-19	TRUE	26
3.84848485	0.00378236	0.00420262	FALSE	9
3.67822967	0.00261831	0.00300461	FALSE	54
4.02688403	3.54E-12	7.75E-12	TRUE	66
4.4496732	1.48E-05	1.92E-05	TRUE	51
5.73059361	5.61E-06	7.41E-06	TRUE	50
4.02867445	7.16E-21	3.86E-20	TRUE	32
5.67829457	4.71E-08	7.67E-08	TRUE	23
4.1746988	2.55E-21	1.78E-20	TRUE	53
4.21445221	0.00046463	0.0005706	TRUE	56

3.91341065	7.37E-14	1.84E-13	TRUE	52
4.25098039	8.24E-14	1.99E-13	TRUE	40
4.03781513	6.20E-18	1.97E-17	TRUE	64
4.40186916	5.74E-20	2.67E-19	TRUE	36

Supplementary Table 2. Summary statistics for regression analysis predicting CCI.

Phecode	Coefficient-sex	Coefficient-age	Coefficient - age:code	Coefficient-code	R^2	pvalue-sex	pvalue-age	pvalue - age:code	pvalue-code
136	0.65170803	0.07683012	0.00076382	-0.0561583	0.34659188	6.06E-10	9.18E-12	2.13E-14	1.22E-06
185	-1.2170954	0.03295062	0.00188891	-0.1696346	0.18836206	0.00044063	0.07112318	1.82E-12	1.95E-13
195.1	0.14581176	0.07421921	0.00066155	-0.0790606	0.14748015	0.11120533	1.58E-12	9.38E-08	2.13E-11
199	0.50227046	0.05491608	0.00078784	-0.0432939	0.23295736	8.68E-13	1.48E-16	7.23E-20	1.98E-07
244.4	0.72928786	0.03277606	0.00085175	-0.0301211	0.27209785	5.41E-40	7.64E-13	7.60E-42	8.41E-07
250	0.25311087	0.11203166	0.00057699	-0.0747665	0.23470845	5.22E-10	1.73E-130	1.25E-22	3.94E-38
250.2	0.25058029	0.11381592	0.00062657	-0.0851737	0.22993946	3.33E-10	7.77E-139	4.92E-24	1.74E-47
250.42	0.42366155	0.06214835	0.00088453	-0.0757565	0.20343491	1.68E-13	1.43E-13	1.34E-35	3.25E-16
261	0.60756224	0.06930618	0.00099971	-0.0816035	0.33268919	8.85E-33	6.23E-23	1.94E-77	8.44E-26
261.4	0.59614404	0.08234301	0.00099297	-0.0939865	0.34954509	1.05E-33	1.84E-30	5.46E-90	3.58E-33
272	0.31598851	0.01574951	0.00118471	-0.0521353	0.21921953	1.81E-29	2.44E-07	7.88E-143	1.23E-32

272.1	0.30783768	0.01831373	0.00119589	-0.0565915	0.22168529	1.25E-29	1.72E-10	7.78E-163	3.08E-44
272.11	0.35874059	0.02806732	0.00107433	-0.0499184	0.20916044	1.31E-23	2.43E-11	1.94E-66	7.88E-19
272.13	0.24610768	0.0526028	0.00107012	-0.0721712	0.23252905	3.72E-06	9.81E-15	1.30E-30	6.53E-19
278	0.27977033	0.01738318	0.00119924	-0.0399576	0.35211207	2.06E-17	2.06E-13	2.93E-237	2.15E-33
278.1	0.26507517	0.03746606	0.00106211	-0.0500658	0.33962057	4.95E-14	3.38E-29	6.02E-160	5.81E-34
285	0.65936643	0.05321796	0.00071175	-0.0355165	0.28582368	5.07E-77	1.43E-56	1.20E-71	2.94E-17
289.4	0.60896021	0.00676081	0.00044615	0.05812349	0.34287669	2.07E-21	0.18789648	6.61E-12	3.12E-19
296	0.53281598	0.04809417	0.00094858	-0.044075	0.34628831	1.11E-39	2.48E-40	3.76E-85	3.62E-17
296.1	0.02719418	0.0547521	0.00139463	-0.0830343	0.36262314	0.77150247	7.76E-10	3.39E-24	3.88E-12
296.22	0.49752673	0.0583585	0.00096487	-0.0552894	0.35345846	4.78E-23	8.73E-42	4.82E-55	4.84E-21
300.9	0.07249106	0.07737533	0.00117438	-0.0917659	0.31683925	0.5233282	3.53E-13	2.09E-13	9.96E-12
306	0.22982941	0.03634598	0.00083715	-0.0380983	0.35869623	2.04E-15	4.04E-34	1.01E-207	8.71E-47
316	0.19319886	0.07621553	0.00108621	-0.0725644	0.32823189	0.01274143	2.74E-25	3.93E-24	4.54E-13

318	0.17274411	0.0398748	0.00097834	-0.0326204	0.28888259	0.00010607	1.03E-17	1.05E-35	1.47E-06
327	0.27745134	0.02599878	0.00094104	-0.0300384	0.34741342	5.38E-11	2.59E-11	1.43E-125	5.74E-10
327.3	-0.0679127	0.04880834	0.00088834	-0.0453768	0.32257971	0.15575158	2.02E-22	8.83E-79	6.49E-16
327.32	-0.0455427	0.0432274	0.00085655	-0.0388217	0.3086265	0.36944127	1.27E-09	1.63E-64	1.86E-08
338.2	0.35785304	0.1194211	0.0007745	-0.1107295	0.25520706	8.90E-11	8.63E-44	2.52E-27	8.21E-34
350.1	0.38072656	0.04160924	0.00080571	-0.0369375	0.33016653	1.33E-06	3.24E-09	1.41E-29	3.12E-06
350.2	0.51018759	0.08844689	0.0003461	-0.0341277	0.33158109	7.57E-16	7.65E-39	3.23E-10	5.62E-06
351	0.36934737	0.01187937	0.00107889	-0.0415537	0.20613458	4.34E-12	0.02578956	2.06E-34	8.47E-08
380.4	0.29603457	0.00431159	0.00122865	-0.0432531	0.34688308	3.52E-08	0.25054454	7.83E-123	1.12E-15
381.1	0.12186198	0.0172657	0.00102824	-0.0321125	0.45125256	0.00721018	2.30E-09	4.85E-123	7.71E-14
389	0.27804228	0.00848033	0.00112695	-0.0330794	0.40028653	1.48E-11	0.00322393	6.01E-198	8.86E-18
401	0.34712562	0.06032393	0.00107572	-0.0925399	0.22131003	7.90E-38	3.22E-111	3.07E-168	1.94E-135
401.1	0.32239731	0.06286607	0.00107182	-0.0955089	0.22774757	2.21E-35	1.51E-134	3.47E-176	1.31E-163

411.4	0.00143098	0.06636191	0.00065584	-0.0515487	0.14351244	0.97308321	4.31E-27	9.22E-13	3.16E-10
416	0.24266997	0.07852242	0.0004899	-0.0491615	0.17832262	1.60E-05	1.94E-37	6.69E-14	1.04E-11
418	0.42340575	0.03922735	0.00092271	-0.0387338	0.30074838	2.28E-46	4.80E-53	2.10E-131	7.29E-26
427	0.3943244	0.03348432	0.00081702	-0.0284488	0.27040654	5.04E-35	1.25E-30	1.50E-117	3.39E-14
427.5	0.30820967	0.0509353	0.00067414	-0.0262037	0.26087743	5.59E-11	3.58E-31	7.15E-32	1.57E-06
427.9	0.52334731	0.04099007	0.00088239	-0.0360591	0.28973139	2.32E-25	3.65E-19	3.72E-40	1.37E-08
459.9	0.29035724	0.04055555	0.00077654	-0.0367438	0.22833552	7.22E-05	5.93E-09	8.41E-23	7.94E-06
465	0.30317808	0.01217126	0.00116902	-0.0344069	0.42341818	6.91E-24	7.30E-10	0	9.03E-37
465.2	0.18851236	0.01690035	0.00115343	-0.0427147	0.39795269	3.35E-06	3.85E-08	3.43E-186	2.57E-26
495	0.2032713	0.02902277	0.00095227	-0.027451	0.4576097	1.09E-07	2.33E-21	1.94E-157	2.90E-14
508	0.2150882	0.07121847	0.00045937	-0.0257971	0.22590114	1.53E-05	2.09E-39	3.23E-16	2.01E-05
512	0.39160321	0.03214072	0.00092054	-0.0337961	0.33621301	1.13E-49	3.82E-46	2.06E-295	7.45E-33
512.1	0.32566379	0.08163241	0.00068319	-0.0520776	0.45111003	1.72E-06	3.78E-26	8.24E-34	6.53E-11

512.2	0.56719804	0.00269342	0.00056099	0.03735339	0.26692044	2.53E-16	0.62988405	5.51E-09	1.94E-05
512.7	0.51136005	0.09733276	0.00056863	-0.0609697	0.27232296	7.52E-46	2.13E-118	3.43E-34	5.47E-33
512.9	0.25608293	0.03722669	0.00075718	-0.0240287	0.2967466	1.04E-12	9.55E-34	1.17E-107	3.26E-10
514	0.4327814	0.10135406	0.00038142	-0.0487608	0.20173864	6.44E-17	5.19E-55	1.85E-07	6.33E-11
530	0.42091139	0.0283581	0.00086407	-0.0231339	0.32945312	8.48E-37	8.75E-20	3.95E-168	2.51E-10
530.1	0.37672142	0.02559237	0.00090657	-0.0247138	0.33482885	2.18E-29	4.94E-16	1.33E-182	1.78E-11
530.11	0.40214932	0.0325333	0.00091996	-0.035009	0.32102239	1.39E-34	6.88E-24	4.49E-192	1.27E-23
530.2	0.59980413	0.25205197	0.00104851	-0.2599751	0.31841906	0.00024345	1.03E-37	1.16E-05	1.59E-30
571.5	0.58321468	0.0468706	0.00124863	-0.0713379	0.31802082	1.09E-21	7.66E-14	2.16E-54	2.29E-19
585	0.16665781	0.10126686	0.00029069	-0.0407601	0.22751985	0.00028018	4.36E-72	5.02E-08	1.14E-09
586.4	-0.796342	0.10413824	0.0007898	-0.1000631	0.18193522	1.26E-06	4.69E-12	1.30E-06	8.23E-10
591	0.94275149	0.03108094	0.00078454	-0.0177979	0.34410504	3.41E-104	1.96E-24	1.64E-81	2.20E-05
594.1	0.12069235	0.03520092	0.00102693	-0.0448154	0.26161609	0.118413	4.32E-07	4.32E-23	2.16E-06

599.5	0.45117259	0.02214833	0.00092052	-0.0258176	0.30272424	2.13E-20	5.03E-07	1.40E-69	3.34E-06
605	-0.110697	0.02803018	0.00129312	-0.080672	0.17679487	0.72362499	0.01651883	2.62E-12	1.83E-07
687.1	0.41294497	0.04109293	0.00102813	-0.0550264	0.33231394	2.44E-20	7.53E-27	2.80E-120	2.22E-31
687.4	0.34186277	0.0293484	0.00081905	-0.0227381	0.27377046	1.26E-12	4.84E-15	3.03E-56	7.61E-06
689	0.3271154	-0.0047281	0.0012036	-0.0401664	0.23745387	6.79E-12	0.24358725	1.06E-98	1.29E-13
704	0.29924259	0.02183664	0.00120333	-0.0617854	0.29499008	1.84E-05	0.00010737	6.96E-57	4.79E-17
733	0.88588587	0.07254698	0.00062177	-0.0552768	0.20299533	8.99E-37	3.15E-37	7.88E-17	2.27E-15
741	0.32267424	0.03468065	0.0010518	-0.050944	0.29249351	2.87E-13	1.91E-18	3.81E-108	2.13E-23
743	0.98648769	0.02706128	0.00084277	-0.0371354	0.18621926	4.80E-92	5.14E-09	3.22E-50	5.90E-10
745	0.28414194	0.02566907	0.00122944	-0.0610717	0.31904006	1.61E-26	1.24E-33	0	2.73E-89
760	0.348592	0.01975726	0.00109892	-0.0398737	0.30364443	7.36E-28	1.33E-14	1.71E-157	4.05E-25
761	0.3341	0.02095084	0.00111104	-0.0433768	0.2907312	4.46E-13	1.37E-07	2.92E-73	4.05E-14
771.1	0.44819658	0.07954079	0.00058201	-0.0421588	0.25732391	5.91E-19	6.10E-50	6.25E-19	1.53E-10

783	0.38881713	0.06822508	0.00058396	-0.0276873	0.41476876	2.36E-24	1.87E-83	4.41E-72	3.33E-12
785	0.52102293	0.02651861	0.0010197	-0.0321999	0.3726903	2.89E-71	8.32E-35	1.77E-250	2.22E-27
789	0.69524975	0.062411	0.00064033	-0.0272881	0.39543249	4.53E-72	3.05E-77	5.17E-80	1.70E-12
790.6	0.44633545	0.03456501	0.00081525	-0.0325441	0.25511127	3.41E-17	9.83E-13	9.02E-43	1.27E-07
798	0.58784477	0.03038112	0.00085179	-0.0232235	0.31575816	3.73E-74	4.14E-26	5.67E-122	2.19E-09
939	0.33210152	0.01873808	0.00101898	-0.0290875	0.40023155	1.95E-20	3.82E-13	2.86E-217	6.87E-19

Supplementary Table 3. Demographics summary of the randomly sampled 100k UW EHR patients and the whole UW EHR cohort.

Cohort	N samples	Age (mean + std)	Sex (female)	Race
University of Washington (whole validation cohort)	844,217	51.59±22.69	451,909(53.53%)	White, 552,554 (65.45%)<br>Asian, 81,868 (9.70%)<br>Unavailable or Unknown, 49468 (5.86%)<br>Black or African American, 37,959 (4.50%)<br>Declined to Answer, 25,979 (3.24%)<br>Black or African-American, 20,759 (2.46%)<br>American Indian or Alaska Native, 7,100 (0.84%)<br>Native Hawaiian or Other Pacific Islander 4,491 (0.53%)<br>American Indian or Native Alaskan 2,921 (0.35%)<br>Native Hawaiian or Pacific Islander 1,969 (0.23%)<br>Mixture (More than one) 59,149 (7.01%)

University of Washington (sampled validation cohort)	100,000	52.23±20.54	54,473(54.49%)	White, 67,203 (69.68%)<br>Asian, 9,794 (10.15%)<br>Unavailable or Unknown, 5,074 (5.26%)<br>Black or African American, 4,480 (4.64%)<br>Declined to Answer, 3,176 (3.29%)<br>Black or African-American 2,811 (2.91%)<br>American Indian or Alaska Native, 787 (0.82%)<br>Native Hawaiian or Other Pacific Islander, 517 (0.54%)<br>American Indian or Native Alaskan, 384 (0.40%)<br>Native Hawaiian or Pacific Islander, 296 (0.31%)<br>Mixture (More than one) 5,478 (5.48%)

Supplementary Table 4. Demographics summary of the randomly sampled 100k UW EHR patients and the whole UW EHR cohort.

phecode	pval	coef	ci_low	ci_high	c-index
136	1.49E-10	-0.2114783	-0.2761821	-0.1467746	0.84772375
185	1.42E-05	-0.1027993	-0.1492224	-0.0563761	0.80500706
195.1	8.73E-06	-0.120369	-0.1734258	-0.0673122	0.84585451
199	4.67E-09	-0.1349106	-0.1800438	-0.0897774	0.83065037
244.4	3.22E-12	-0.1248476	-0.1599668	-0.0897284	0.83356971
250	5.62E-07	-0.105717	-0.1471247	-0.0643093	0.81770209
250.2	1.27E-20	-0.1258305	-0.152318	-0.0993429	0.83250644
250.42	7.07E-09	-0.1259937	-0.16865	-0.0833375	0.86082666
261	1.12E-08	-0.1811891	-0.2433732	-0.1190051	0.84035141
261.4	1.78E-12	-0.1554092	-0.1986099	-0.1122085	0.85173708
272	3.44E-06	-0.0785036	-0.1116474	-0.0453599	0.81267112
272.1	1.82E-22	-0.1204084	-0.1446101	-0.0962066	0.82303717
272.11	6.98E-10	-0.1184213	-0.1560606	-0.080782	0.83040326
272.13	2.82E-06	-0.1175253	-0.166708	-0.0683425	0.87108014
278	1.02E-10	-0.1621762	-0.2113548	-0.1129975	0.82497293

278.1	9.37E-13	-0.1727683	-0.2201975	-0.1253392	0.82579338
285	9.69E-17	-0.1092189	-0.1349836	-0.0834543	0.84913619
289.4	1.75E-14	-0.1905307	-0.2392332	-0.1418281	0.82990815
296	3.07E-07	-0.1072809	-0.1483549	-0.066207	0.81200454
296.1	2.07E-05	-0.1765791	-0.2578651	-0.0952931	0.84548927
296.22	3.66E-07	-0.0893854	-0.1238315	-0.0549392	0.85687421
300.9	8.78E-06	-0.2068249	-0.2980157	-0.1156341	0.83009688
306	2.21E-12	-0.0983872	-0.1258557	-0.0709188	0.8588313
316	6.67E-08	-0.1185925	-0.1616385	-0.0755464	0.80333553
318	1.38E-13	-0.1313157	-0.1661024	-0.096529	0.80433002
327	3.34E-10	-0.1503259	-0.1972272	-0.1034246	0.83373297
327.3	2.27E-08	-0.1370444	-0.1850918	-0.0889969	0.82534178
327.32	1.24E-13	-0.1528251	-0.1932349	-0.1124154	0.84167498
338.2	2.53E-45	-0.1908827	-0.2173623	-0.1644031	0.87027828
350.1	1.72E-09	-0.1676949	-0.2222691	-0.1131207	0.86542247
350.2	2.39E-06	-0.0881016	-0.1247056	-0.0514976	0.86582385
351	1.34E-06	-0.1208975	-0.1699115	-0.0718835	0.84234161

380.4	4.87E-08	-0.1372041	-0.1864932	-0.0879151	0.86885888
381.1	1.94E-05	-0.2403197	-0.3505725	-0.130067	0.86748453
389	1.95E-07	-0.1150115	-0.1583256	-0.0716974	0.8472802
401	2.43E-14	-0.1109002	-0.1394043	-0.0823961	0.82016957
401.1	1.20E-50	-0.1258029	-0.1422768	-0.109329	0.83753524
411.4	2.05E-15	-0.1124855	-0.1402575	-0.0847135	0.8324278
416	4.56E-06	-0.1823915	-0.2603783	-0.1044046	0.86433701
418	1.54E-21	-0.1486113	-0.179169	-0.1180535	0.83762818
427	1.46E-07	-0.0907487	-0.1245779	-0.0569196	0.8278567
427.5	5.45E-06	-0.1238168	-0.1771914	-0.0704423	0.84718335
427.9	6.04E-07	-0.1388944	-0.1934516	-0.0843372	0.84788643
459.9	1.10E-06	-0.1372233	-0.192417	-0.0820297	0.85998947
465	1.92E-11	-0.1423054	-0.1838609	-0.10075	0.84773644
465.2	9.01E-06	-0.1669586	-0.2406672	-0.0932499	0.84865955
495	2.05E-10	-0.1558844	-0.2039419	-0.1078269	0.84500562
508	2.43E-05	-0.118876	-0.1740704	-0.0636816	0.82419275
512	8.30E-19	-0.1533077	-0.1872373	-0.119378	0.81904691

512.1	3.24E-08	-0.1851387	-0.2507831	-0.1194942	0.86545506
512.2	5.33E-11	-0.272098	-0.3533776	-0.1908185	0.88293792
512.7	1.11E-30	-0.1422293	-0.1664383	-0.1180203	0.85567693
512.9	1.87E-35	-0.1908498	-0.2209503	-0.1607493	0.85550344
514	1.39E-11	-0.1273574	-0.1642876	-0.0904272	0.83827337
530	6.46E-06	-0.0894124	-0.1282635	-0.0505613	0.81188346
530.1	1.85E-05	-0.089047	-0.1298018	-0.0482922	0.81294623
530.11	8.49E-20	-0.1197689	-0.1455459	-0.0939919	0.83676466
530.2	1.27E-05	-0.1798925	-0.260654	-0.0991311	0.80741618
571.5	5.91E-12	-0.2000121	-0.2569754	-0.1430488	0.80212028
585	5.67E-10	-0.1338415	-0.1761568	-0.0915262	0.84628285
586.4	1.28E-08	-0.2337033	-0.3142146	-0.1531921	0.88270892
591	1.11E-10	-0.1070631	-0.1395885	-0.0745377	0.85054392
594.1	8.88E-06	-0.1519267	-0.2189511	-0.0849023	0.83706179
599.5	1.69E-08	-0.1184546	-0.1596093	-0.0772998	0.84292594
605	2.34E-06	-0.1583298	-0.2240505	-0.0926091	0.85191533
687.1	1.22E-17	-0.1587678	-0.1951572	-0.1223783	0.85645916

687.4	3.43E-07	-0.1094753	-0.1515611	-0.0673895	0.82078569
689	2.69E-13	-0.1561263	-0.197992	-0.1142606	0.86220173
704	9.25E-06	-0.2070002	-0.2985025	-0.1154978	0.86711392
733	1.53E-05	-0.1309966	-0.1903693	-0.0716238	0.88567482
741	1.15E-06	-0.118124	-0.1657154	-0.0705327	0.8183197
743	2.42E-07	-0.1264397	-0.1744314	-0.078448	0.84914063
745	5.59E-21	-0.1153473	-0.1394043	-0.0912902	0.83337709
760	6.12E-11	-0.1105318	-0.1436525	-0.0774112	0.81497028
761	6.97E-07	-0.1169593	-0.1631547	-0.0707639	0.81214219
771.1	5.21E-09	-0.1113381	-0.1487023	-0.073974	0.8497934
783	7.17E-07	-0.0890861	-0.1243118	-0.0538604	0.82631999
785	1.04E-25	-0.1469115	-0.1743797	-0.1194434	0.83508909
789	2.69E-19	-0.1291276	-0.1573091	-0.1009462	0.82576321
790.6	1.04E-13	-0.1569106	-0.1982685	-0.1155528	0.86987098
798	1.16E-33	-0.148538	-0.1726139	-0.1244621	0.8376273
939	3.43E-07	-0.1036229	-0.14346	-0.0637859	0.83361752

Supplementary Table 5. ANOVA table extending analysis to digital phenotype PC3-5.

Results for PC3 (explained variance ratio 8.96%)

variable	df	sum_sq	mean_sq	F	PR(>F)
**site**	11.0	16988.010787	1544.364617	10894.949185	0.000000e+00
**age**	1.0	998.040012	998.040012	7040.821251	0.000000e+00
**sex2**	1.0	667.095139	667.095139	4706.121571	0.000000e+00
**PC1**	1.0	241.854405	241.854405	1706.197761	0.000000e+00
**PC5**	1.0	16.329449	16.329449	115.198521	7.363674e-27
**PC7**	1.0	11.588512	11.588512	81.752875	1.568661e-19

Results for PC4 (explained variance ratio 8.46%)

variable	df	sum_sq	mean_sq	F	PR(>F)
**site**	11.0	7676.652258	697.877478	2974.502226	0.000000e+00
**PC1**	1.0	36.235794	36.235794	154.444662	1.966504e-35
**sex2**	1.0	14.747986	14.747986	62.859054	2.243082e-15
**PC8**	1.0	14.144831	14.144831	60.288280	8.270424e-15
**PC5**	1.0	9.424816	9.424816	40.170571	2.337124e-10
**age**	1.0	7.734305	7.734305	32.965251	9.409070e-09

Results for PC5 (explained variance ratio 4.73%)

variable	df	sum_sq	mean_sq	F	PR(>F)
**site**	11.0	2745.436485	249.585135	1728.046518	0.000000e+00
**age**	1.0	183.711204	183.711204	1271.956786	7.722664e-277
**PC4**	1.0	51.134901	51.134901	354.041471	7.643082e-79
**PC1**	1.0	27.381346	27.381346	189.579555	4.299238e-43
**PC2**	1.0	16.479654	16.479654	114.099776	1.280723e-26
**PC3**	1.0	2.626707	2.626707	18.186466	2.004802e-05

Supplementary Table 6. Mapping tables.

Phenotypes	Phecode	h2	Median onset age
ADHD	313.1	0.55928184	13.2291667
Abnormal_Spine_Curvature	737.3	0.3018074	21.8541667
Acne	706.1	0.54177841	30.8958333
Acquired_Coagulation_Defect	286.4	0.32458128	59.9791667
Acquired_Hypothyroidism	244.4	0.25036868	65.2708333
Acquired_Limb_Deformities	736	0.00422273	56.1041667
Acquired_Retinal_Defects	362	0.30342514	76.2916667
Acquired_Visual_Disturbances	368	0.4731518	62.1666667
Acute_Bronchitis	483	0.4313531	59.5416667
Acute_Renal_Failure	585.1	0.00328995	64.6875
Acute_Sinusitis	464	0.3564863	52.9791667
Acute_Upper_Respiratory_Infection	465	0.20162013	44.9375
Adjustment_Disorder	304	0.31013778	51.9791667
Adrenogenital_Disorder	255.3	1	19.8125
Allergic_Rhinitis	476	0.45558223	47.0625

Alopecia	704.1	0.23794009	52.9791667
Alveolar_Disease	504	0.47	63.9791667
Alzheimers_Disease	290.11	0.34526316	82.1458333
Aneurysm	442.1	0.57304714	70.9791667
Aniridia	753	1	62.7291667
Anophthalmia_Mircopthalmia	753	1	62.7291667
Anterior_Eye_Anomaly	753	1	62.7291667
Anxiety_Phobic_Disorder	300.13	0.26198495	55.9583333
Aplastic_Anemia	284	0.08189935	55.2083333
Appendiceal_Disease	540	0.16880765	47.4583333
Aromatic_Amino_Acid_Metabolism_Disorder	270.1	1	45.9791667
Arterial_Embolism_Thrombosis	444	0.00333582	65.4583333
Asthma	495	0.413015	48.375
Atherosclerosis	440	0.27761222	69.5416667
Atopic_Contact_Dermatitis	939	0.44367123	52.5625
Autism	313.3	1	10.0833333
Autoimmune_Polyglandular_Disorder	259	1	49.3333333

Balanced_Translocation	758.1	1	7.35416667
Benign_Bone_Connective_Tissue_Neoplasm	215	0.21768801	54.9375
Benign_Breast_Neoplasm	610.4	0.2875629	52.8541667
Benign_Colon_Neoplasm	208	0.13511053	66.6666667
Benign_Digestive_Neoplasm	211	0.5056619	63.9791667
Benign_Oropharyngeal_Neoplasm	210	0.22961183	61
Benign_Prostastic_Hyperplasia	600	0.00874628	70.9791667
Benign_Skin_Neoplasm	216	0.44789019	58.5625
Benign_Uterine_Neoplasm	218.2	0.14352765	47.7708333
Biliary_Tract_Disease	575.8	0.21867746	59.9791667
Bipolar_Disorder	296.1	0.4138892	47.9791667
Bladder_Cancer	189.21	0.08682608	72.5416667
Bladder_Disorder	596	0.07863395	63.9791667
Brain_Damage	348.9	0.2263792	59.0625
Branched_Chain_Amino_Acid_Metabolism_Disorder	270.1	1	45.9791667
Breast_Cancer	174.11	0.22740077	61.7916667
Breast_Disorder	613.9	0.41379434	56.9791667

CNS_Cancer	191.1	0.12	43.9791667
Carbuncle_Furuncle	686.1	0.42414579	50.375
Cardiac_Conduction_Disorder	426	0.3375	72.4375
Cardiac_Congenital_Anomaly	747.1	0.30676741	19.9791667
Cardiac_Dysrhythmia	427.5	0.17965809	65.9583333
Cardiomyopathy	425.1	0.20360742	62.7083333
Carditis	420	0.00503279	59.9791667
Cataplexy_Narcolepsy	347	0.37	55.4583333
Cataract	366	0.23389101	74.8958333
Cellulitis	681	0.13550789	60.6875
Cerebrovascular_Disease	433	0.19417304	70.8541667
Cervical_Cancer	180.1	0.15391268	51.9791667
Chondrodystrophy	359.1	1	40
Choroidal_Disorders	363	0.6	70.6666667
Chronic_Glomerulonephritis	580.12	0.39	37.7083333
Chronic_Kidney_Disease	585.3	0.21076384	62.9791667
Chronic_Liver_Disease	573	0.29523383	59.9166667

Chronic_Pruritus	698	0.22698779	62.6041667
Chronic_Sinusitis	475	0.29962414	54.9791667
Chronic_Tonsillitis_Adenoiditis	474.2	0.28729808	7.60416667
Chronic_Upper_Respiratory_Infection	479	0.37544876	56
Circulating_Enzyme_Deficiency	697	0.00638181	57.3333333
Colorectal_Cancer	153	0.16188926	64.5416667
Conduct_Disorder	312	0.47340198	11
Congenital_Aphakia	753.1	1	16.2708333
Congenital_Hypogammaglobulinemia	279.11	0.615	54.9791667
Congenital_Hypothyroidism	244.5	0.4424979	8.5
Congenital_Pigmentary_Anomaly	694.2	0.24444787	62.7708333
Conjunctival_Disorders	372	0.17326832	67.1041667
Connective_Tissue_Cancer	170.2	0.00472316	55.1458333
Connective_Tissue_Infection	709.7	0.00472316	53.5833333
Copper_Metabolism_Disorder	275.2	1	49
Corneal_Disorders	364	0.15919207	69.625
Cranial_Nerve_Disorder	352	0.24919145	60.6458333

Cystic_Fibrosis	499	0.39414212	17.9791667
Cystitis_Urethritis	592.2	0.28232444	53.2916667
Deep_Vein_Thrombosis	452.2	0.22483741	64.5625
Degenerative_Dementia	290.1	0.00108166	81.9791667
Depression	296.22	0.34566227	53.9791667
Developmental_Delay	264.9	0.38653645	5.64583333
Deviated_Nasal_Septum	470	0.48875109	55.6875
Disease_of_the_Female_Reproductive_Organs	619	0.33754	48.4166667
Disorder_of_the_Vestibular_System	386	0.45333333	66.625
Disorders_of_Fatty_Acid_Oxidation	277	1	38.9791667
Disorders_of_Straight_Chain_Amino_Acid_Metabolism	270.1	1	45.9791667
Disorders_of_Urea_Cycle_Metabolism	270.21	1	51.8125
Diverticulosis_Diverticulitis	562.1	0.01233876	68.4583333
Dyschromia	694.2	0.24444787	62.7708333
Ear_Infection	381.11	0.29853824	7.97916667
Eating_Disorder	305.2	0.48561295	36.1875
Edwards_Syndrome	758.1	1	7.35416667

Ehlers_Danlos_Syndrome	756.3	1	23.9166667
Ellis_Van_Creveld_Syndrome	756.5	1	58.75
Emphysema_COPD	496.1	0.44513737	70.2708333
Endocrine_Cancer	194	0.28	25.2083333
Epidermolysis_Bullosa	691	1	47.6041667
Epilepsy_Related_Disorders	345.12	0.4729964	17.75
Esophageal_Cancer	150	0.25	66.6041667
Esophageal_Disease	530	0.23317511	60.3333333
Esophagitis	530.1	0.19539576	59.9791667
Eustachian_Tube_Disorder	381.2	0.52285795	49.9791667
External_Ear_Disorders	380	0.44244332	68.0833333
Extrapyramidal_Abnormal_Movement_Disorders	333	0.13189399	64.3958333
Eye_Infection	369.5	0.40840606	50.4791667
Eye_Inflammation	371.3	0.41511455	70.75
Eyelid_Disorders	374	1	71.4166667
Familial_Dysautonomia_Related_Conditions	752.2	1	5.97916667
Fasciitis	712	0.34986551	57.3125

Female_Genital_Cancer	184.1	0.00259297	62.9791667
Female_Genital_Congenital_Anomaly	751.1	1	6.35416667
Food_Allergy	930	0.61451321	9.41666667
Fragile_X_Syndrome	758	1	7.20833333
Functional_Digestive_Disorder	564	0.13173912	56.9166667
Fungal_Infection	110.2	0.25805877	48.9791667
Galactosemia	271.9	1	13.9375
Gastritis_Duodenitis	535	0.12483038	58.9791667
Gastrointestinal_Infection	8	0.745	56.4375
Gastrointestinal_Ulcer	531.2	0.26694362	65.9791667
General_Bacterial_Infection	41	0.2196654	57.4791667
General_Dementia	290.1	0.00108166	81.9791667
General_Ear_Disorder	388	0.2433403	67.3125
General_Hypertension	401.1	0.30880097	66.875
General_Osteochondropathy	732	0.4913862	14.6458333
General_Spondylosis_Spine_Disorder	721.1	0.25151315	66.7916667
General_Thrombocytopenia	287.3	0.712181	61.7083333

General_Viral_Infection	79.9	0.42038973	21.9791667
Generalized_Digestive_Cancer	159	0.00798863	64.4583333
Generalized_Lipidoses	277.51	1	55.7291667
Genetic_Anomolies_of_Leukocytes	288	1	60.9791667
Gestational_Pregnancy_Related_Disorder	646	0.03500042	31.4583333
Glaucoma	365	0.33822992	72.9166667
Goiter	240	0.26019461	60.0833333
Gout_Related_Crystal_Arthropathies	274.2	0.30900579	74.2291667
Graves_Disease	242.1	0.79	55.3541667
Haemophilia_Related_Diseases	286.5	1	62.9791667
Hair_Related_Disease	704.1	0.23794009	52.9791667
Hearing_Loss	389	0.34419155	66.0625
Hemangioma_Lymphangioma	228	0.37722881	61.9375
Hemiplegia	342	0.00130766	58.9166667
Hepatobiliary_Cancer	155	0.23	63.4375
Hereditary_Blepharophimosis	374	1	71.4166667
Hereditary_Corneal_Dystrophy	364.5	1	72.9791667

Hereditary_Dental_Anomaly	520.1	1	53.9375
Hereditary_Elliptocytosis	282.9	1	26.375
Hereditary_Fructose_Intolerance	271.9	1	13.9375
Hereditary_Harartoses	741.5	1	66.8958333
Hereditary_Hemoglobin_Disease	282.8	0.59690807	45.9375
Hereditary_Polycythemia_Vera	200.1	1	69.2708333
Hereditary_Progessive_Muscular_Dystrophy	359.1	1	40
Hereditary_Sensory_Neuropathy	356	1	66.9791667
Hereditary_Spastic_Paraplegia	334.1	1	49.9166667
Hereditary_Stomatocytosis	282.9	1	26.375
Hernia	550.5	0.14187713	60.7916667
Herpes_Simplex	54	0.29924031	48.9791667
Herpes_Zoster	53	0.12667042	67.75
Hirschsprungs_Disease	750.21	1	7.58333333
Hirsutism	704.2	0.69	39.1041667
Hodgkins_Disease	201	0.00301466	42.9791667
Homocystinuria	270.11	1	55.7083333

Huntingtons_Chorea	357	0.10736189	63.9791667
Hyper_IgM_Immunodeficiency	279.11	0.615	54.9791667
Hyperaldosteronism	255.12	0.4	64.0416667
Hyperchylomicronemia	272.14	1	60.2291667
Hypotension	458.9	0.24	65.9791667
Ichthyosis_Congenita	691.1	1	47.7083333
IgA_Immunodeficiency	279.11	0.615	54.9791667
IgM_Immunodeficiency	279.11	0.615	54.9791667
Impulse_Control_Disorder	312.3	0.51909165	15.2083333
Indeterminate_Sex_Pseudohermaphroditi sm	751.1	1	6.35416667
Infestation	132	0.47237037	40.5416667
Inflammatory_Spondylopathies	715	0.36182779	58.125
Influenza	481	0.42087012	59.9791667
Inner_Ear_Disorder	386	0.45333333	66.625
Intellectual_Disability	315.3	0.4732918	14.9583333
Intestinal_Cancer	153.2	0.00534984	66.9791667
Jaw_Disease	526.9	0.204748	59.9791667

Keratosis	702.1	0.34373327	71.9791667
Kidney_Cancer	189.11	0.04017051	63.9791667
Kidney_Infection	590	0.2209515	54.5833333
Klinefelters_Syndrome	758.1	1	7.35416667
Klippel_Feil_Syndrome	754	1	59.5
Lacrimal_System_Disorder	375	0.19675058	68.0833333
Learning_Disorder	315.1	0.2384184	11.625
Lipodystrophy	277.51	1	55.7291667
Lipoma	214.1	0.03787306	60.1875
Lipoprotein_Deficiency	277.51	1	55.7291667
Localized_Adiposity	278.3	0.4844525	51.9791667
Long_QT_Syndrome	426.8	1	62.7708333
Lung_Cancer	165.1	0.00213514	69.625
Lupus_Erythematosus	695.42	0.50388733	43.9791667
Lymphatic_Disorder	450	0.21494737	64.9583333
Lymphoid_Leukemia	204.1	0.24	28.4166667
Lysosomal_Storage_Disease	277.51	1	55.7291667

Macular_Degeneration	362.29	0.43633333	81.8125
Male_Genital_Cancer	187.1	0.13407055	62.5208333
Male_Genital_Congenital_Anomaly	751.12	0.9	4.8125
Malnutrition	260	0.27124886	59.9791667
Marfan_Syndrome	758	1	7.20833333
Medullary_Cystic_Kidney_Disease	751.21	1	54
Melanoma	172.11	0.07427463	65.8333333
Menopausal_Disorder	627	0.47	58.5
Menstrual_Disorder	626.13	0.42256684	40.5208333
Migraine	340	0.26537065	46.9791667
Mixed_Hyperlipidemia	272.13	0.61590644	63.9791667
Mood_Disorder	296	0.36950052	52.7916667
Motor_Neuron_Disease	334.2	1	56.3333333
Multiple_Epiphyseal_Dysplasia	756.5	1	58.75
Multiple_Myeloma	204.4	0.00301466	64.9791667
Multiple_Sclerosis_Other_Demyelinating_Disease	335	0.162925	49.9791667
Muscle_Ligament_Disorder	728.2	0.24078728	14.0208333

Myasthenia_Gravis	358.1	0.821	56.8958333
Mycobacterial_Disease	31	0.467519	62.6458333
Myeloid_Leukemia	204.2	0.00301466	55.9791667
Myocardial_Infarction	411.2	0.01391189	68.5833333
Myotonic_Muscular_Dystrophy	359.1	1	40
Nail_Disease	703	0.21949427	73.5
Neonatal_Disease	656	0.3195742	25.0208333
Nerve_Root_Plexus_Disorders	353	0.33883745	56.3541667
Neural_Tube_Defect	752	0.70175	6.08333333
Neurofibromatosis	199.4	0.52744645	29.6041667
Nezelofs_Syndrome	279.1	1	49.4583333
OCD	300.3	0.41108016	42.7708333
Obesity	278.1	0.40629848	55.1458333
Ocular_Musculoskeletal_Disorder	376	0.3527453	64.9583333
Optic_Disc_Disorder	377	0.695	56.9791667
Optic_Neuritis_Neuropathy	377.3	0.55652371	60.6875
Oral_Soft_Tissue_Disease	729	0.11340278	64.3333333

Osteoarthritis	740.9	0.24809381	67.4583333
Osteogenesis_Imperfecta	756.5	1	58.75
Osteomyelitis	710.19	0.10993342	59.8125
Osteopetrosis	756.5	1	58.75
Osteoporosis	743.11	0.14433597	71.0208333
Other_Infectious_Diseases	136	0.24118308	54.9791667
Other_Inherited_Amino_Acid_Metabolic_Disorder	270.2	1	13.9791667
Other_Viral_Hepatitis	70	0.0006925	54.0208333
Ovarian_Cancer	184.11	0.20070831	63.0833333
PTSD	300.9	0.37157619	47.1458333
Pancreatic_Cancer	157	0.212	66.4791667
Pancreatic_Disease	577	0.00155506	52.3958333
Pancytopenia	284.1	0.19	59.9791667
Paranoia	301	0.49982491	51.0208333
Parasitic_Infection	134	0.1050896	51.9791667
Parathyroid_Disorder	252	0.44793415	64.2083333
Parkinsons_Disease	332	0.24539833	73.9791667

Pataus_Syndrome	758.1	1	7.35416667
Peripheral_Nerve_Disorder	357	0.10736189	63.9791667
Peripheral_Vascular_Disease	443.9	0.27802585	71.875
Pernicious_or_B12_Deficiency_Anemia	281.12	0.32881535	66.7291667
Personality_Disorder	301	0.49982491	51.0208333
Phenyketonuria	270.12	1	6.97916667
Phlebitis_Thrombophlebitis	451	0.01043445	61.1666667
Phosphorus_Metabolism_Disorder	275.53	1	59.4791667
Photodermatitis	938.1	0.51579221	65.125
Pituitary_Disorder	253	0.0062405	46.5
Pleuritis	507	0.09181226	65.9791667
Pneumonia	480	0.19657153	63.2708333
Poliomyelitis	324	0.00492542	62.1875
Polycystic_Kidney_Disease	751.21	1	54
Polycystic_Liver_Disease	750.22	1	14.7291667
Polymorphonuclear_Neutrophils_Disorders	288	1	60.9791667
Polyostotic_Fibrous_Dysplasia_of_Bone	756.5	1	58.75

Porphyrin_Metabolic_Disorder	277.1	1	59.3541667
Prader_Willi_Syndrome	758	1	7.20833333
Prion_Disease	324.1	0.55	73.7916667
Prostate_Cancer	185	0.23123644	70.9791667
Protozoan_Infection	8	0.745	56.4375
Psoriasis_Related_Disorders	696.4	0.34938644	55.9791667
Pubertal_Dysfunction	259.3	0.68014275	15.3333333
Pulmonary_Embolism_Infarction	415.11	0.00544174	63.375
Pulmonary_Heart_Disease	415.2	0.00043504	64.375
Pure_Hyperglyceridemia	272.12	1	59
Purine_Pyrimidine_Metabolic_Disorder	277.2	1	16.7916667
Purpura_Other_Hemorrhagic_Conditions	287	0.00063456	59.9791667
Radiation_Sensitivity	938	0.585	67.0416667
Rectal_Anal_Disorder	565	0.0417914	54.7083333
Refraction_Accomodation_Disorders	367	0.34930897	63.625
Refsums_Disease	356	1	66.9791667
Renal_Glycosuria	250.5	1	48.3958333

Respiratory_System_Cancer	165.1	0.00213514	69.625
Rheumatic_Heart_Disease	428.1	0.55365441	70.9791667
Rheumatoid_Arthritis_Related_Conditions	714.1	0.43014648	61.9791667
Rosacea	695.3	0.28644285	61.0625
STI	90	0.27449875	48.875
Salivary_Gland_Disease	527	0.40299525	62.5208333
Sarcoidosis	697	0.00638181	57.3333333
Schizophrenia_Related_Psychosis	295.3	0.38950328	61.9791667
Seborrheic_Dermatitis	690.1	0.37170086	63.2708333
Secondary_Arthropathy	740.3	0.62886708	72.9166667
Secondary_Hereditary_Retinal_Dystrophy	362.7	0.6	63.9791667
Septic_Arthritis	711.1	0.19251475	63
Septicemia	38	0.19646148	61.9791667
Severe_Combined_Immunodeficiency	277.2	1	16.7916667
Sexual_Dysfunction	302	0.28354093	54.9791667
Sjogrens_Syndrome	709.2	0.54	62.1875
Skin_Infection	686	0.31212944	60.6041667

Sleep_Apnea	327.32	0.21963204	64.9791667
Sleep_Disorder	327	0.28428332	60.8958333
Speech_Language_Disorder	315.2	0.36412082	6.25
Spinal_Muscular_Atrophy	334.2	1	56.3333333
Spinal_Stenosis	720	0.50576468	70.75
Spinocerebellar_Ataxia	334.1	1	49.9166667
Staphylococcus_Aureus_Infection	41.11	0.19	56.2291667
Stomach_Cancer	151	0.01	67.4166667
Streptococcal_Infection	41.2	0.00217228	58.4166667
Substance_Abuse	316	0.31629883	44.5833333
Syndrome_with_Pervasive_Congenital_Anomalies	313.3	1	10.0833333
Synovium_Tendon_Bursa_Disorder	727	0.26617606	61.3541667
Thyroid_Cancer	193	0.53	55
Thyroiditis	245	0.63507469	53.875
Thyrotoxicosis	242	0.20690308	58.4583333
Tics_Tourettes	313.2	0.44743934	12.8541667
Tongue_Disease	529	0.6204765	63.5

Tuberculosis	10	0.0018469	52.125
Tuberous_Sclerosis	759	1	10.7291667
Turners_Syndrome	758.1	1	7.35416667
Tympanic_Membrane_Disorders	384	0.29840147	51.2291667
Type_II_Diabetes_Mellitus	250.2	0.32868981	65.9791667
Type_I_Diabetes_Mellitus	250.1	0.43715471	50.9791667
